# On the necessity of dissecting sequence similarity scores into segment-specific contributions for inferring protein homology, function prediction and annotation

**DOI:** 10.1186/1471-2105-15-166

**Published:** 2014-06-02

**Authors:** Wing-Cheong Wong, Sebastian Maurer-Stroh, Birgit Eisenhaber, Frank Eisenhaber

**Affiliations:** 1 Bioinformatics Institute (BII), Agency for Science, Technology and Research (A*STAR), 30 Biopolis Street, #07-01, Matrix, Singapore 138671, Singapore; 2 School of Biological Sciences (SBS), Nanyang Technological University (NTU), 60 Nanyang Drive, Singapore 637551, Singapore; 3 Department of Biological Sciences (DBS), National University of Singapore (NUS), 8 Medical Drive, Singapore 117597, Singapore; 4 School of Computer Engineering (SCE), Nanyang Technological University (NTU), 50 Nanyang Drive, Singapore 637553, Singapore

**Keywords:** Sequence homology, Protein domain library, Hidden Markov model, Sequence similarity search, Non-globular protein sequence segment, Automated protein function annotation, Similarity score dissection

## Abstract

**Background:**

Protein sequence similarities to any types of non-globular segments (coiled coils, low complexity regions, transmembrane regions, long loops, etc. where either positional sequence conservation is the result of a very simple, physically induced pattern or rather integral sequence properties are critical) are pertinent sources for mistaken homologies. Regretfully, these considerations regularly escape attention in large-scale annotation studies since, often, there is no substitute to manual handling of these cases. Quantitative criteria are required to suppress events of function annotation transfer as a result of false homology assignments.

**Results:**

The sequence homology concept is based on the similarity comparison between the structural elements, the basic building blocks for conferring the overall fold of a protein. We propose to dissect the total similarity score into fold-critical and other, remaining contributions and suggest that, for a valid homology statement, the fold-relevant score contribution should at least be significant on its own. As part of the article, we provide the DissectHMMER software program for dissecting HMMER2/3 scores into segment-specific contributions. We show that DissectHMMER reproduces HMMER2/3 scores with sufficient accuracy and that it is useful in automated decisions about homology for instructive sequence examples. To generalize the dissection concept for cases without 3D structural information, we find that a dissection based on alignment quality is an appropriate surrogate. The approach was applied to a large-scale study of SMART and PFAM domains in the space of seed sequences and in the space of UniProt/SwissProt.

**Conclusions:**

Sequence similarity core dissection with regard to fold-critical and other contributions systematically suppresses false hits and, additionally, recovers previously obscured homology relationships such as the one between aquaporins and formate/nitrite transporters that, so far, was only supported by structure comparison.

## Background

The modus operandi of the modern day sequence homology concept [1,2] is founded on two inductively proven implications: (i) the inference of evolutionary history from sets of homologous protein sequences (e.g. 1964, fibrinopeptides [3]; 1967, cytochrome c [4]) to build believable phylogenetic trees [5,6]; (ii) the inference of homology for functionally uncharacterized sequences with high sequence similarity to proteins with characterized structure and/or function through the trinity of sequence-structure-function relationship (e.g., in 1967, lactalbumin model was built using the X-ray coordinates of lyzosome where the two sequences are concluded to be homologous for being 35% identical [7]; in 1986, angiogenin is homologous to pancreatic ribonuclease where the X-ray structure of the latter is known [8,9]).

In both proofs, there are some crucial, yet problematic assumptions [10]. In the first implication, it requires the antecedent that the sequences are homologous (the event of common evolutionary origin p), then, as a consequence, the sequences are expected to be high in similarity (event q; thus, we have *p* → *q*). Whereas this first implication appears quite acceptable (as well as the contrapositive form ¬ *q* → ¬ *p*, low sequence similarity would rather imply absence of homology though evolution might have erased sequence similarity), the second one is by far not obvious. In the proof of the second implication where structure/function similarity is concluded from high sequence similarity (actually *q* → *p*), the conserved key amino acids in the uncharacterized sequence for concluding similarity to the structure/function of the well-studied protein need to be those that correspond to the hydrophobic patterns responsible for the 3D structure formation and the residues critical for binding/catalysis/etc. To note, in both cases of inductive proofs, the proteins under scrutiny were soluble, globular proteins of limited size without non-globular segments.

Thus, homology has the precise meaning of “having a common evolutionary origin” but it also carries the loose meaning of “possessing sequence similarity or being matched”. In addition, homology between sequences is always a hypothesis while similarity, being a measurable fact, can be attributed to either chance, convergent evolution or common ancestry [11-13]. In other words, high sequence similarity is a necessary but insufficient condition for concluding homology.

Fortunately, sequence similarity by chance can be eliminated via stringent statistical criteria like E-value cutoffs in Blast [14] or HMMER-based [15,16] sequence searches. Nevertheless, the statistical cutoff does not help in reversing the conditional statement *p* → *q* into *q* → *p* since the issue of distinguishing between convergent evolution and common ancestry among hits of high similarity is non-trivial. As a guide, similarities to any types of non-globular segments (coiled coils, low complexity regions, transmembrane regions, long loops, etc. where either positional sequence conservation is the result of a very simple, physically induced pattern or rather integral sequence properties are critical) are pertinent sources for mistaken homologies [10,17-19]. Although this issue has been mentioned even in early work [2], regretfully, these considerations regularly escape attention in large-scale annotation studies since, often, there is nothing to substitute manual handling of these cases. Quantitative criteria are required to suppress events of function annotation transfer as a result of false homology assignments. Our previous work has shown that the exclusion of undesirable signal peptides (SPs) and simple transmembrane helices (TMs) in protein domain models can suppress many unrelated sequence hits and even reveal true homologies that, otherwise, would have disappeared in the noise [10,19-21].

Standard alignment tools (e.g. BLAST [14], HMMER [15,16,22]) and domain libraries (e.g. SMART [23,24], Pfam [25,26]) have become the obligatory components of many modern-day automated annotation pipelines for detecting homology and, hence, to infer the functions of many unknown sequences accumulating in the relentlessly growing sequence databases. But these automated packages operate strictly in the similarity space with preset score or, equivalently, E-value cutoffs. Thus, statistically significant similarities of any aligned pieces following as the program outputs are declared as homologies without any alternative consideration of convergence cases. The latter operation *q* → *p* is a non-equivalent converse statement of the original proof *p* → *q*. Indeed, this is the bane of current sequence search approaches that, frequently, lead to wrongful protein function predictions or annotations, especially when one attempts to extrapolate very deep into sequence space [27-29].

To alleviate the abovementioned issue, we reiterate that the working principle of the sequence homology concept is based on the similarity comparison between the structural elements, the basic building blocks for conferring the overall fold of a protein which in turn characterizes its biological function [30]. To note, the issue of alignment segmentation into blocks of higher quality more relevant for structure, fold and function conservation has been discussed widely in context of multiple alignment generation, fold recognition and threading [31-34]. Therefore, a viable approach for improving the existing sequence searches is to dissect each total alignment into two types of segments. The first class is suggestive of structured, essential components providing a conserved, complex hydrophobic/hydrophilic sequence pattern (termed “fold-relevant”, “fold-critical” or “structured” segments) possibly complemented by further, function-critical positions. The other group of segments includes all types of non-globular segments, very long loops and other elaborations in 3D structures, etc. that are not under the same fold/function conservation evolutionary pressure (termed “remnant” segments) [17]. The purpose is to independently re-evaluate the respective two score sums for statistical significance, subsequently. As a necessary condition to be considered as a valid hit, the total score of fold segments should either be more statistically significant than the score sum of remnant segments or, minimally, be statistically significant on its own.

To further emphasize, the concept of a globular domain has a deeply-rooted notion where it implies a sequence segment (or several of those, a domain does not need to be contiguous) having an independent tertiary structure (i.e., an autonomous hydrophobic core), it folds and melts autonomously. Its sequence evolves as a unit in phylogeny [30]. The unsettling thing is that a sizeable number of domain models in protein domain libraries often represent something else, not a globular domain in the sense as described above. The model might consist of several globular domains or contain non-globular additions. Since the sequence homology-based annotation transfer in the case of low sequence identity is applicable only for the single globular domain, some type of model dissection becomes intuitively important. One can either go via the work-intensive route of creating new, elementary domain model libraries or, alternatively, follow the path of score dissection with regard to the contributing sequence segments. Generally speaking, the idea of score dissection is more generic and is applicable to any existing sequence-based methods (whether Blast-based [14], HMMER-based [15,16,22] or profile-profile-based [35,36]) as long as one can reconstruct the alignment scores from the various parameterization of the search algorithms. In addition, score dissection does not require the original algorithms to be modified.

In this work, we achieved four main objectives. First, we created an algorithm and the software tool DissectHMMER (provided as supplement to this MS [37]) that can re-compute the scores of HMMER2 and HMMER3 and assign the respective contributions to predefined query – domain model alignment segments. We were able to achieve good replication of the log-odd scores/E-values generated by both HMMER2 and HMMER3 across all the seed sequences in SMART and Pfam domains. Second, we show the usefulness of this tool in case studies where dissecting the alignment scores into fold-critical and remnant contributions (using PDB/DSSP information) enables us to identify false hits that are statistically significant for the total HMM model and, at the same time, we could elucidate previously insignificant true hits among the truly false ones.

Third, to generalize the dissection framework to domains without PDB/DSSP representation, the quality score based on alignment quality was introduced. Out of 635 SMART and 5876 Pfam domains with structures, 537 SMART and 4771 Pfam domains were found to be enriched with structural residues in their high-quality segments. This was more than 80% of the statistically testable cases. Thus, the quality score is justifiable surrogate for estimating fold-related and remnant segments in domain models. Importantly, this and similar criteria can be applied to segmenting HMM models in domain libraries without having the domain alignments to be re-edited or the HMMER searches to be rerun.

Finally, the application of the dissection framework (using quality score) on the seed alignments of SMART and Pfam domains gave an average positive concordance rates of almost 100% and a negative one of less than 1%. The latter implies that almost all of the seed sequences were recognized correctly as true hits. Meanwhile, the dissection of alignment results from searches against the UniProt/SwissProt for these SMART and Pfam domains returned average false-positive rates of less than 1% but average false-negative (FN) rates of 7.63% (SMART) and 4.86% (Pfam). The latter presents an opportunity to recover previously obscured homologous relationship between the FN hits and its associated domain model. Filtering for domain models that have exceptionally high error rates also allows finding those cases where reconsidering the seed alignment might be useful.

## Results

### Methodology for the reconstruction of HMMER2 and HMMER3 scores

**Figure 1:**
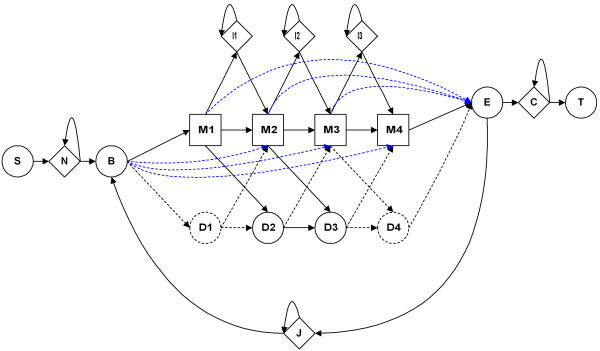
**Scheme of an HMM protein domain model.** This figure is adapted after Figure one in [39]. Blue lines show transitions for which local model parameters are not delivered by hmmconvert for HMMER3.

In the current implementation of the HMMER packages (HMMER2 [15,38] and HMMER3 [16,39]), a single, total log-odd score is returned for each domain-to-sequence alignment. Fundamentally, each score is composed of two types of contributions: the positional scores (made between the HMMER emitted sequence and the hit sequence) and the position-invariant scores (Figure 1 designed after Figure one in [39]).

The positional scores are composed from a series of emission (at each state; e.g. M1/D1/I1) scores and transition (state-to-state; e.g. M1- > I1, M1- > M2) scores where M/D/I are match, delete and insert states. In the case of the invariant scores, they account for the fixed transition entry scores (e.g. N- > B, B- > M) and exit scores (M- > E, E- > C) for each domain-to-sequence alignment. These are added to the positional scores to give the final log-odd score of the alignment. As a rule, these positional and position-invariant components are retrievable from the respective HMM model files provided with domain libraries. Then, the reconstruction of the HMMER scores follows the straightforward arithmetic computations as described in equation (1) (see Methods).

In fact, the score reconstruction has already been applied on HMMER2 glocal (align a complete model to a subsequence) and global (align a complete model to a full sequence) outputs in one of our earlier works [10]. Therefore, the score reconstruction procedure should logically be directly applicable to the HMMER3 domain-to-sequence alignments.

However, two issues ensue to complicate the straightforward procedure. First, the current implementation of HMMER3 [39] lacks support of the glocal/global search mode. Hence, local alignments are to be expected since there is no way to enforce glocal/global alignments. For the cases of seed sequences that are closely related to the domains, the local alignments will somewhat resemble the glocal/global alignment generated by HMMER2 and the HMMER2 score reconstruction can still achieve good replication results. But for many cases of fragmented local alignments, their reconstruction will have less precision in comparison due to the following issues.

This problem stems from the exclusion of certain invariant score parameters during the conversion of HMMER3 model files to HMMER2 format. Regretfully, the conversion is necessary to export the HMMER3 null model parameters (as part of the log-odd score parameters) since they are embedded in the HMMER3 program code, the second major issue. In contrast, the HMMER2 null model parameters are already captured in their model files. To note, the HMMER3 software suite only allows for model conversion (via hmmconvert -2) from the HMMER3 local model to the HMMER2 glocal/global model. In the process, only the first HMMER state (B- > M_1_, B- > D_1_; see Figure 1) and last state (M_K_- > E, D_K_- > E; see Figure 1) were kept while the other transition log-odd scores (e.g. B- > M_2..K-1_ shown by blue lines in Figure 1) were excluded from the converted HMMER3 model files since these parameters are not part of a global model. Therefore, the reconstruction of HMMER3 local alignment score is bound to suffer some estimation errors inherently due to the unavailability of these parameters for the straightforward summing.

In hindsight though, the estimation is not detrimental to the overall accuracy of HMMER3 score reconstruction as demonstrated by the subsequent section. It is in fact only slightly less accurate than the HMMER2 reconstruction. Only in cases where HMMER3 returns heavily fragmented alignments, the reconstruction error becomes noticeable; yet, it is still sufficiently small to not interfere in the significance analysis of the segmental subscores.

In this work, a program – DissectHMMER, was written to compute the reconstructed score relative to pre-defined alignment segments using the alignment (the HMM output) and the HMM model file as inputs independent on the HMMER suite version used (2 or 3). The algorithmic detail is described in the Methods section. The code is provided as Additional file 1 (as zip archive and at the accompanying WWW site [37]).

### Reproducibility and error estimation of the reconstructed HMMER log odd scores

To summarize, the score calculation in the various HMMER versions is a complicated routine with some parts not explicitly documented in the literature. Besides algorithmic assumptions, numerical issues such as rounding errors also play a role. Thus, it cannot be expected that the reconstructed scores exactly match the scores reported by HMMER but it is close enough for the purpose of reconstructing the segmental contributions to the total score.

To test the score reconstruction workflow, the seed alignments from SMART version 6 and Pfam release 27 were used. In comparison to SMART, the current Pfam library is about 12 times larger and, hence, the rigor of the scores reproduction was truly being tested in this case. In total, 735 SMART domains (excluding 73 domains with less than 5 seed sequences) and 12121 Pfam domains (excluding 2711 domains with less than 5 seed sequences) were examined.

For each domain alignment, the HMMER model is first built (using hmmbuild with null2 option off) and, then, it is searched against (using hmmsearch -F) the same set of seed sequences. For each seed sequence, the alignments reported are considered true hits. By this constraint, both HMMER2 and HMMER3 share the same search space and, hence, the alignments generated by both are expected to be similar (if not identical). Next, the HMMER log-odd scores for the total alignment were reconstructed as described in Methods (see equations (1 and 2)).

**Figure 2:**
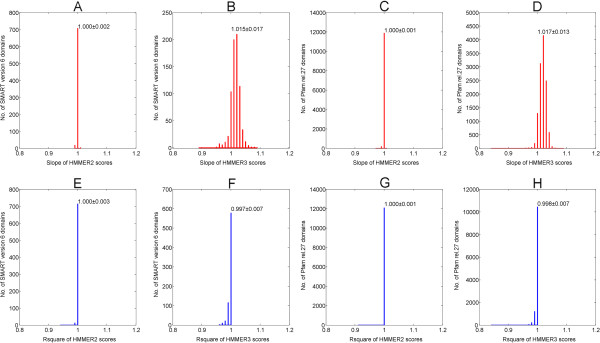
**Regression analysis output (slope **$\hat{\beta }$** and coefficient of determination*r***^**2**^**) for both SMART (version 6) and Pfam (release 27) domains.** Figure **A** and **B** depict the histograms of the slopes $\hat{\beta }$ for the original versus reconstructed scores for SMART domains calculated for HMMER2 and HMMER3 respectively while Figure **C** and **D** depict the histograms of the slopes $\hat{\beta }$ for the Pfam domains. The HMMER2 results exhibit high reproducibility at an average $\hat{\beta }$ of 1.000 ± 0.001 (SMART) and 1.000 ± 0.002 (Pfam) while HMMER3 results also show good, though slightly worse reproducibility with average $\hat{\beta }$ of 1.015 ± 0.017 (SMART) and 1.017 ± 0.013 (Pfam). Figures **E**, **F**, **G** and **H** shows the corresponding histograms for the goodness of fit, in terms of *r*^2^. Similarly, the HMMER2 reconstruction exhibits excellent fit at an average *r*^2^ of 1.000 ± 0.003 (SMART) and 1.000 ± 0.007 (Pfam). HMMER3 reconstruction closely followed at an average *r*^2^ of 0.997 (SMART) and 0.998 (Pfam) over a slightly larger variation of 0.007 (SMART/Pfam). In hindsight, all values of $\hat{\beta }$ and *r*^2^ converges to one with little variation and this implies that the reconstruction workflow for HMMER2/3 scores are highly reproducible.

Once this computation was completed for all seed sequences of a given domain, linear regression analysis was performed against the original scores (see equations (3 and 4) in Methods). The regression analysis output, in terms of slope ($\hat{\beta }$) and coefficient of determination (*r*^2^) as goodness of fit, is plotted for both SMART (version 6) and Pfam (release 27) domains in Figure 2. Figure 2A and B depict the histograms of the slopes $\hat{\beta }$ for the original versus reconstructed scores for SMART domains calculated for HMMER2 and HMMER3, respectively, while Figure 2C and D depict the histograms of the slopes $\hat{\beta }$ for the Pfam domains. Generally speaking, the HMMER2 results exhibit high reproducibility at an average $\hat{\beta }$ with an ideal value of 1.000 (SMART/Pfam) with small standard deviations of 0.001 (SMART) and 0.002 (Pfam). In comparison, HMMER3 results also show good, though slightly worse reproducibility with average $\hat{\beta }$ of 1.015 ± 0.017 (SMART) and 1.017 ± 0.013 (Pfam).

**Figure 3:**
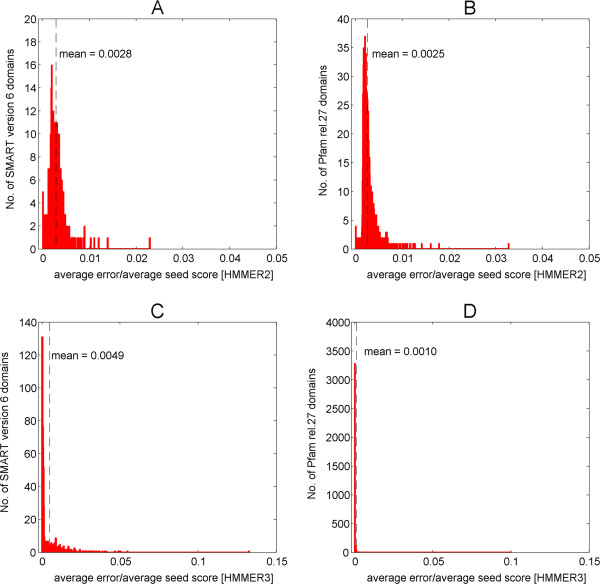
**Relative error estimates per SMART/Pfam domain.** Figures **A**, **B** and **C**, **D** show the histograms of the relative errors for the HMMER2 and HMMER3 results and the SMART and PFAM domain databases respectively. The average reconstruction errors by HMMER2 were 0.0028 (SMART) and 0.0025 (Pfam) and mostly well below the 0.01 margin (or 1% of the average seed score per domain) as depicted by the vertical dashed lines. Likewise, the average reconstruction errors attributed by HMMER3 are 0.0049 and 0.0010 for SMART and Pfam domains respectively (See vertical dashed lines). They are well below the 0.05 line (or 5% of the average seed score per domain). Generally speaking, the relative errors tend being dwarfed by their respective domain-wise alignment scores for all seed sequences.

The goodness of fit, in terms of coefficient of determination (*r*^2^), for the original versus reconstructed HMMER2 and HMMER3 scores are depicted in Figure 2E, F, G and H respectively as histograms. Again, the HMMER2 reconstruction exhibits excellent fit at an average *r*^2^ of 1.000 (SMART/Pfam) and small standard deviations of 0.003 (SMART) and 0.007 (Pfam). HMMER3 reconstruction closely followed at an average *r*^2^ of 0.997 (SMART) and 0.998 (Pfam) over a slightly larger variation of 0.007 (SMART/Pfam). Taken together, the general trend where all values of $\hat{\beta }$ and *r*^2^ converges to one with little variation, implies that the reconstruction workflow for HMMER2/3 scores are highly reliable and reproducible. The reconstruction works well for the relatively small SMART library as well as for the huge Pfam library.Next, the relative error estimates per SMART/Pfam domain were examined (Figure 3, see equations (5, 6, 7 and 8) in Methods). To note, the scores generated for various seed sequences of one domain are quite similar to each other in the case of HMMER2, mostly, because the glocal mode enforces alignments of similar length. In the case of HMMER3, the alignments are often (almost) identical with those in the HMMER2 case. Yet, the alignments for a large number of many other seed sequences are heavily fragmented. Since we are interested in assessing the error of reconstruction over the representative domain score and not over each individual alignment fragment where, especially, the assignment of gap scores to the individual fragment scores by HMMER3 is difficult to recover as discussed above, we rather compare the total error of reconstruction for the seed sequence – domain alignment with the sum of scores for all the seed – domain alignment fragments reported. Therefore, we estimate the error for each domain as ratio between the sum of deviations between original and reconstructed score for each seed sequence on the one hand and the sum of original scores for each seed sequence on the other hand. Figure 3A, B and C, D show the histograms of the relative errors for the HMMER2 and HMMER3 results and the SMART and PFAM domain databases, respectively. The majority of the reconstruction errors by HMMER2 are well below the satisfactory 0.01 margin (or 1% of the average seed score per domain) and at an average of 0.0028 (SMART) and 0.0025 (Pfam) as depicted by the vertical dashed lines. Similarly, the reconstruction errors attributed by HMMER3 are well below the 0.05 line (or 5% of the average seed score per domain). The average relative errors are about 0.0049 and 0.0010 for SMART and Pfam domains, respectively (see vertical dashed lines). As a general trend, the relative errors tend being dwarfed by their respective domain-wise alignment scores for all seed sequences.

Taken together, the results show that the reconstruction recovers the original score within a few percent at worst. Since we wish to make a qualitative conclusion whether a certain alignment segment of the total query sequence – domain alignment makes a substantial or even overwhelming contribution to the total score, the reconstruction algorithm with all errors taken into consideration appears well suited for the purpose.

**Figure 4:**
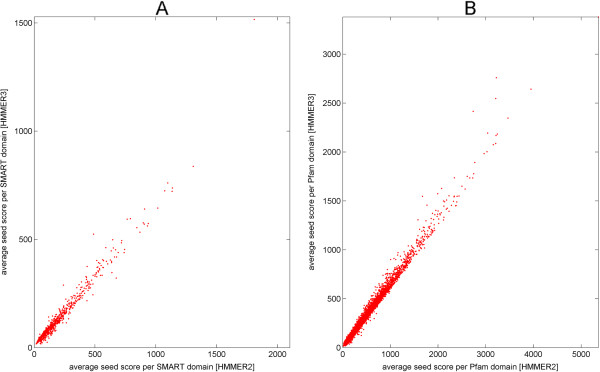
**HMMER2 versus HMMER3 average domain score (averaged over all seed sequences) for SMART (version 6) and Pfam (release 27).** Figure **A** shows the comparison of HMMER2 versus HMMER3 domain scores for 735 (out of 808) SMART domains while Figure **B** shows the comparison for 12121 (out of 14831) Pfam domains. As a trend, the HMMER3 scores are smaller than the HMMER2 scores but strongly correlated (the goodness of fit *r*^2^ is 0.9692 for y = 0.6785x in the case of SMART and 0.9867 for y = 0.6629x in the case of Pfam).

This large scale study of seed sequence scores also allows comparing some aspects of HMMER2 and HMMER3 program behaviors. Figure 4 shows the HMMER2 versus HMMER3 score averaged over all seed sequences for each domain plotted for all domains (Figure 4A SMART, Figure 4B Pfam). As a trend, the HMMER3 scores (y-axis) are clearly smaller than the HMMER2 scores (x-axis). They are strongly correlated (the goodness of fit *r*^2^ is 0.9692 for y = 0.6785x in the case of SMART and 0.9867 for y = 0.6629x in the case of Pfam) but not equivalent. To note, this work was not planned as a comparative study between the two tools and we strived as much as possible to focus on conclusions supported by either program.

### Dissection of sequence alignments accentuates homology evidence in true hits while deemphasizes false hits

The idea of dissecting a HMM score into several segments of a larger alignment stems from the observation that the influence of well conserved, truly homologous alignment segments on the score can be overwhelmed by score contribution from spurious alignment extensions. In our previous work [10,19], we have shown that the score enhancements from aligning non-relevant SP/TM hydrophobic stretches can create the appearance of high scores and significant E-values of alignments between unrelated sequences.

**Figure 5:**
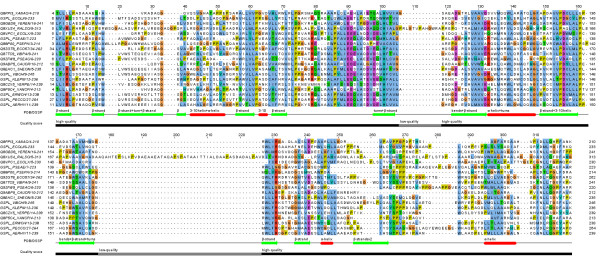
**Segmentation by DSSP and by quality score for an example alignment.** We show the seed alignment of PF05134.8 (T2SL, type II secretion system protein L). Below the alignment, two segmentations are presented. Red and green segment in the upper line are assigned labels “H, B, E, G, I, T, S” the DSSP [40] file for the structure 1 W97 (chain L) and together represent the respective fold-relevant part. In the lower line, the segmentation is based on alignment quality giving rise to black (fold-relevant) and grey (remnant) segments.

At the same time, it is well accepted that structural elements are the basic building blocks for conferring the overall fold of a protein which in turn characterizes its biological function. Therefore, for the purpose of inferring homology, one should evaluate the score of the structural, fold-relevant segments independently from the score associated with remnant segments. Figure 5 shows an example of such a segmentation highlighting the fold-relevant alignment pieces (based on the seed alignment of PF05134.8 T2SL). Furthermore, as a necessary condition to be considered as a true hit, the structural, fold-relevant score should either be more statistically significant than the score for other segments or, at least, it should be statistically significant on its own. The postmortem dissection of the alignment can provide additional insights beyond what a standard single total score/E-value could, as illustrated through a selected, validated set of 13 hits (some of them are true and and others are actually false) found by 8 Pfam domains (PF01298.13 Lipoprotein 5, PF04814.8 HNF-1_N, PF05134.8 T2SL, PF09110.6 HAND and PF10390.4 ELL, PF00004.24 AAA, PF00106.20 adh_short and PF01226.12 Form_Nir_trans) as listed in Table 1.

**Table 1:** Examples of validated false hits from 5 Pfam domains (PF01298.13 Lipoprotein5, PF04814.8 HNF-1 N, PF05134.8 T2SL, PF09110.6 HAND, PF10390.4 ELL) and validated true hits from 3 Pfam domains (PF00004.24 AAA, PF00106.20 adh_short, PF01226.12 Form_Nir_trans)

**Domain name**	**Hit name**	**HMMER version**	**Total score (E-value)**	**Fold-critical score (E-value**_**1**_**)**	**Remnant score (E-value**_**2**_**)**	**Ratio of E-value**_**1**_**: E-value**_**2**_
PF01298.13 Lipoprotein5	1.sp|O60841|IF2P_HUMAN (Eukaryotic translational initialization factor 5B)	HMMER2	−183.8 (3.1)	−164.6 (7.6e-1)	−7.6 (6.7e-6)	1.1e + 5
		HMMER3	30.1 (6.7e-8)	−2.9 (5.8e + 4)	22.8 (1.0e-3)	5.8e + 7
Domain length: 979	2.sp|Q05D44|IF2P_MOUSE (Eukaryotic translational initialization factor 5B)	HMMER2	−184.6 (3.3)	−150.5 (2.7e-1)	−24.9 (2.4e-5)	1.1e + 4
		HMMER3	26.5 (8e-7)	4.4 (3.9e + 2)	33.9 (4.6e-7)	8.5e + 8
PDB:3V8U|B	3.sp|Q5RDE1|IF2P_PONAB (Eukaryotic translational initialization factor 5B)	HMMER2	−185.0 (3.4)	−137.5 (1.0e-1)	−33.2 (4.5e-5)	2.2e + 3
		HMMER3	28.6 (1.8e-7)	−2.9 (5.8e + 4)	22.2 (1.5e-3)	3.9e + 7
	4.sp|Q7XTT4|NUCL2_ORYSJ (Nucleolin 2)	HMMER2	−190.8 (5.2)	−130.5 (6.1e-2)	−50.2 (1.6e-4)	3.8e + 2
		HMMER3	13.2 (8.2e-3)	−5.0 (2.0e + 5)	14.5 (3.3e-1)	6.1e + 5
PF04814.8 HNF-1_N (Hepatocyte nuclear factor 1)	5.sp|Q6PDK2|MLL2_MOUSE (Histone-lysine N-methyltransferase 2D)	HMMER2	−70.2 (1.5)	−45.6 (1.1e-2)	−15.4 (2.5e-5)	4.4e + 2
		HMMER3	24.5 (5.1e-6)	0.0 (2.9e + 4)	32.3 (4.1e-6)	7.1e + 9
Domain length: 250	6.sp|P41046|CORTO_DROME (Centrosomal/chromosomal factor)	HMMER2	−75.5 (4.4)	−55.3 (7.6e-2)	−6.1 (3.9e-6)	2.0e + 4
		HMMER3	23.0 (1.6e-5)	0.0 (2.9e + 4)	32.9 (2.8e-6)	1.0e + 10
PDB:1IC8|B	7.sp|Q54RP6|DHKL_DICDI (Hybrid signal transduction histidine kinase L)	HMMER2	−75.6 (4.5)	−52.5 (4.3e-2)	−6.9 (4.5e-6)	9.6e + 3
		HMMER3	32.6 (1.7e-8)	0.0 (2.9e + 4)	47.7 (8.3e-11)	3.5e + 14
PF05134.8 T2SL (Type II secretion system protein L)	8.sp|Q8VHG2|AMOT_MOUSE (Angiomotin)	HMMER2	−81.4 (4.5)	−69.3 (7.6e-1)	10.5 (6.1e-6)	1.3e + 5
Domain length: 321		HMMER3	18.2 (1.8e-5)	8.4 (3.6e + 1)	28.2 (3.5e-5)	1.0e + 6
PDB:1 W97|L						
PF09110.6 HAND (Chromatin remodeling factor ISW1a)	9.sp|P19338|NUCL_HUMAN (Nucleolin)	HMMER2	−39.7 (2.1)	−40.8 (2.6)	16.7 (3.6e-5)	7.2e + 4
PDB:2Y9Z|A		HMMER3	23.3 (2.7e-5)	3.7 (2.3e + 3)	22.1 (5.0e-3)	4.6e + 5
PF10390.4 ELL (RNA polymerase II elongation factor)	10.sp|P34103|PK4_DICDI (Protein kinase 4)	HMMER2	−70.7(3.7e-2)	−49.4 (2.9e-3)	−13.0 (3.9e-5)	7.4e + 1
Domain length: 139		HMMER3	94.5 (2.5e-27)	0.0 (9.2e + 3)	99.8 (5.5e-27)	1.7e + 30
PDB:2E5N|A						
PF00004.24 AAA (ATPase family associated with various cellular activities)	11.sp|P51394|CHLI_PORPU (Magnesium-chelatase subunit ChII)	HMMER2	−27.2 (1.8)	38.5 (2.2e-6)	−48.2 (1.4e + 2)	1.6e-8
Domain Length: 252		HMMER3	11.3 (1.1e-1)	22.4 (3.1e-3)	6.0 (3.1e + 2)	1.0e-5
PDB:1LV7|A		HMMER3	5.6 (5.9)	26.4 (1.9e-4)	−2.9 (1.4e + 5)	1.4e-9
PF00106.20 adh_short (Short chain dehydrogenase)	12.sp|Q9UXR8|HEM1_METKA (Glutamyl-tRNA reductase)	HMMER2	−49.7 (1.7e-1)	13.7 (1.1e-5)	−54.6 (9.0e-1)	1.2e-5
Domain length: 230		HMMER3	23.0 (7.9e-6)	43.1 (1.5e-9)	−6.4 (5.3e + 5)	2.8e-15
PDB:3MJC|B						
PF01226.12 Form_Nir_trans (Formate/nitrate transporter)	13.sp|Q9ATM0|TIP12_MAIZE (Aquaporin TIP 1–2)	HMMER2	−109.7 (1.3e-1)	−47.5 (1.2e-4)	−45.3 (9.4e-5)	1.3
Domain length: 366						
PDB:3KCU|E						

The segmentation of domain models is based on PDB/DSSP information.

In retrospect, all hit examples (see Table 1, column 2) were retrieved from the results of HMMER2 (glocal-mode) and HMMER3 when searched against the SwissProt/UniProt sequence database (see later in the text for the general results of this test). To note, the hmmsearch option ‘nobias’ in HMMER3 was turned off to increase the search sensitivity (ability to detect true hits) as stated in the manual [39]. For example, the true hit glutamyl-tRNA reductase (HEM1_METKA) was not detected by HMMER3 when the ‘nobias’ option was turned on. Next, the representative structures for the Pfam domains were obtained by searching against PDB FASTA database for the most significant hit with E-value < 0.1 using the global HMM model (HMMER2) for maximum model coverage.

Then, the structural residues (carrying “H, B, E, G, I, T, S” labels in the DSSP files) were retrieved from the corresponding DSSP annotations [40] with the purpose of dissecting each domain alignment into its fold-related/remnant segments so that the final singular fold-related and remnant scores with respect to the hits can be derived using the score reconstruction procedure from the preceding section. Also, all the hits except for TIP12_MAIZE were found by both HMMER2 and HMMER3 (see column 3), although the HMMER3 returned only fragmented alignments which offered only partial coverage with respect to the domain models (see supplementary website [37] for alignments). The statistical significance E-value cutoff for the evaluation was 0.1.

Based on a collective view of the standard HMMER output scores/E-values in Table 1 (column 4), the hits produced HMMER2 E-values of between 3.7e-2 to 5.2 and between 2.5e-27 to 1.1e-1 via HMMER3. At an E-value cutoff of 0.1, the overwhelming majority of the hits would be considered false based on HMMER2, yet true by HMMER3. And it would be hard-pressed to tell the differences based on the standard total alignment HMMER score/E-value alone.

**Figure 6:**
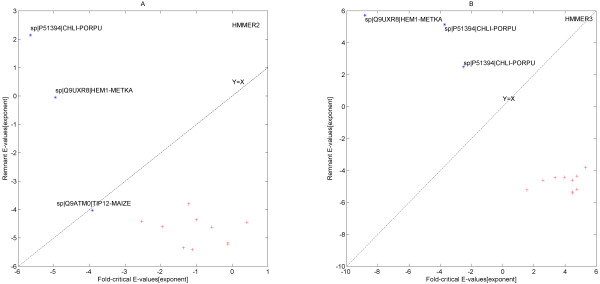
**HMMER2 versus HMMER3 average domain score (averaged over all).** When the fold-critical and remnant scores (see Table 1, columns 5 and 6) were considered, the distinction between the true and false hits becomes apparent. The Y = X margin depicts two regions: above is where the fold-critical E-values were smaller than the reminant E-values and below as vice-versa. As a general trend, the fold-related scores of hits IF2P_HUMAN, IF2P_MOUSE, IF2P_PONAB, NUCL1_ORYSJ, MLL2_MOUSE, CORTO_DROME, DHKL_DICDI, AMOT_MOUSE, NUCL_HUMAN and PK4_DICDI (see red points) were much smaller than the remnant scores indicating that they are spurious hits and their corresponding fold-related E-values spans from 2.9e-2 to 2.6 (HMMER2) and 3.6e + 1 to 2.0e + 5 (HMMER3) against the more significant remnant segments’ E-values ranges of 3.6e-6 to 1.0e-4 (HMMER2) and 5.5e-27 to 3.3e-1 (HMMER3). In contrast, the fold-related scores were larger than the remnant scores for hits CHLI_PORPU, HEM1_METKA (see blue points). For TIP12_MAIZE (see blue point), the difference between its fold-related and remnant scores was marginal. The corresponding fold-related E-values of 1.2e-4 to 2.2e-6 (HMMER2) and 3.1e-3 to 1.5e-9 (HMMER3) were more significant than the remnant segments’ E-values of 9.4e-5 to 1.4e + 2 (HMMER2) and 3.1e + 2 to 5.3e + 5 (HMMER3).

However, once the fold-critical and remnant scores (see Table 1, columns 5 and 6) were considered, the distinction between the true and false hits becomes apparent as depicted in Figure 6. As a general trend, the fold-related scores of hits 1 to 10 (IF2P_HUMAN, IF2P_MOUSE, IF2P_PONAB, NUCL1_ORYSJ, MLL2_MOUSE, CORTO_DROME, DHKL_DICDI, AMOT_MOUSE, NUCL_HUMAN, PK4_DICDI) were vastly smaller than the remnant scores indicating that they are spurious hits. The corresponding fold-related E-values spans from 2.9e-2 to 2.6 (HMMER2) and 3.6e + 1 to 2.0e + 5 (HMMER3) against the more significant remnant segments’ E-values ranges of 3.6e-6 to 1.0e-4 (HMMER2) and 5.5e-27 to 3.3e-1 (HMMER3).

In contrast, the opposite trend was observed for hits 11 and 12 (CHLI_PORPU, HEM1_METKA) where the fold-related scores were larger than the remnant scores. For hit 13 (TIP12_MAIZE), the difference between its fold-related and remnant scores was marginal. The corresponding fold-related E-values of 1.2e-4 to 2.2e-6 (HMMER2) and 3.1e-3 to 1.5e-9 (HMMER3) were more significant than the remnant segments’ E-values of 9.4e-5 to 1.4e + 2 (HMMER2) and 3.1e + 2 to 5.3e + 5 (HMMER3). Thus, the latter three hits are rather true homologies in the segment representing the protein fold.

Furthermore, to investigate the difference in magnitudes between the fold-critical and remnant E-values, their ratios (see Table 1, column 7) were taken. A small ratio (<<1) is indicative that the fold-related component is more significant than its remnant counterpart and, hence, its overall sequence similarity gravitates towards homology. On the other hand, a large ratio is suggestive of spurious sequence similarity. At a ratio of 1, both fold-related and remnant segments’ components are on-par. As such, with the range of ratios between 7.4e + 1 to 1.3e + 5 (HMMER2) and between 4.6e + 5 to 1.7e + 30 (HMMER3), hit 1 to 10 are to be considered as false hits. And with ratios between 1.6e-8 to 1.3 (HMMER2) and between 2.8e-15 to 1.0e-5 (HMMER3), hits 11 to 13 are to be labeled as true hits.

**Figure 7:**
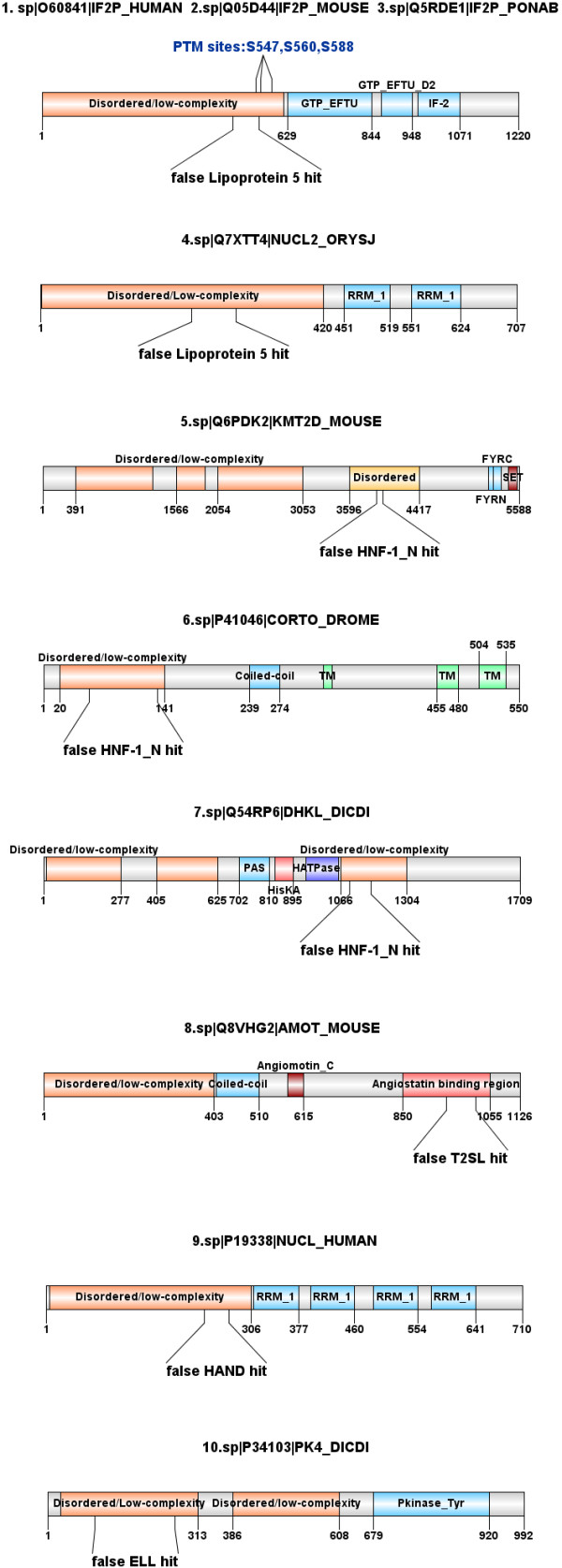
**Domain architectures of the 10 false (false-positive) hits.** The domain architectures of 5 Pfam domain models (*PF01298.13 Lipoprotein5, PF05134.8 T2SL, PF09110.6 HAND, PF10390.4 ELL*) revealed that the 10 hits (1:IF2P_HUMAN, 2:IF2P_MOUSE, 3:IF2P_PONAS, 4:NUCL2_ORYSJ, 5:KMT2D_MOUSE, 6:CORTO_DROME, 7:DHKL_DICDI, 8:AMOT_MOUSE, 9:NUCL_HUMAN, 10:PK4_DICDI) are falsely associated to the respective domain models through a significant non-structural segment which is typically low-complexity and disordered.

For the alleged false hits (rows 1–4 in Table 1), the sequence architecture analysis was performed [41-43] and their false associations with the domains is justified as follows (see Figure 7, HMMER2/3 alignments are available at the associated WWW site [37]). The model Lipoprotein 5 (PF01298.13, row 1) can be represented by the transferring-binding protein B (TbpB) from various bacteria. TbpB is part of the transferring receptor and it is an outer membrane protein that is anchored to membrane via a lipidated N-terminus segment [44]. In contrast to the model, IF2P_HUMAN, IF2P_MOUSE and IF2P_PONAB are translation initialization factors which are essentially cytoplasmic proteins from various eukaryotes while NUCL1_ORYSJ is a plant nucleolin which binds RNA in the nucleus. These diverse proteins were related spuriously to the model via an N-terminal disordered/low-complexity segment with remnant segment’s E-values of 6.7e-6 to 1.6e-4 (HMMER2) and 4.6e-7 to 3.3e-1 (HMMER3). For the translation initialization factors, this linker segment contains multiple phosphorylation sites [45]. Separately, another unrelated domain model HAND (PF09110.6, row 4), a chromatin remodeling factor [46], hits the nucleolin (NUCL_HUMAN) again, albeit human, on the N-terminal disordered/low-complexity segment with E-values of 3.6e-5 (HMMER2) and 5.0e-3 (HMMER3).

Next, the model HNF-1 N (PF04814.8, row 2) describes the N-terminus of the homeobox-containing transcription factor HNF-1 (Hepatocyte nuclear factor 1). The latter contains a dimerization sequence and an acidic region which is implicated in transcription activation [47]. In contrast, the diversely different false hits MLL2_MOUSE, CORTO_DROME and DHKL_DICDI are a methyltransferase, a chromosomal protein and a kinase respectively. They are related to the HNF-1 model merely via a small stretch of N- or C-terminal disordered segments with E-values of 3.9e-6 to 2.5e-5 (HMMER2) and 8.3e-11 to 4.1e-6 (HMMER3).

Meanwhile, the model T2SL (PF05134.8, row 3) describes protein L, an inner membrane protein of the bacterial type II secretion system that serves as a critical link between the cytoplasmic and the periplasmic part of the Eps-system [48]. In contrast, the mouse angiomotin (AMOT_MOUSE) is involved in angiogenesis and regulates the action of the angiogenesis inhibitor angiostatin [49,50]. The angiostatin-binding linker segment of the angiomotin made a false association to this bacterial domain model with remnant segments’ E-values of 6.1e-6 (HMMER2) and 3.5e-5 (HMMER3).

Finally, the model ELL (PF10390.4, row 5) is a RNA polymerase II elongation factor that regulates the polymerase II [51]. Yet, the hit PK4_DICDI, a protein kinase of slime mold, is related to the model through a small stretch of disordered/low-complexity linker with segmental E-values of 3.9e-5 (HMMER2) and 5.5e-27 (HMMER3).

**Figure 8:**
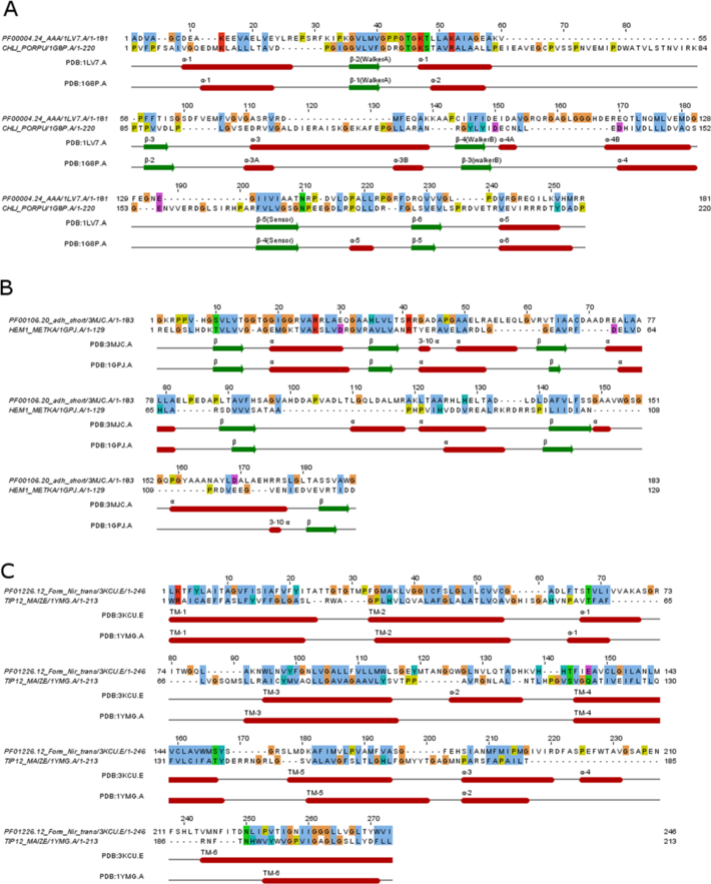
**Structural alignments between representative structures of domain model and hit sequence for the 3 true (false-negative) hits.** The original E-values of these 3 hits (**A**. *CHLI_PORPU,***B***. HEM1_METKA*, **C**. *TIP12_MAIZE*) were insignificant against the Pfam domain models (*PF00004.24 AAA, PF00106.20 adh_short, PF01226.12 Form_Nir_trans)*. However, their structural E-values were nevertheless significant (E < 0.1). Indeed, the structural alignments of representative structures between domain models and hits showed that their RMSD values were between 3.2 to 3.91 and over their full-length sequences. This indicated that the domain model and the associated hit sequences were indeed homologous to each other.

For the alleged true hits, the justification of sequence similarity between the hit and domain model is best shown by fold similarity, especially for cases of distant homologs (indicated by their large E-values) where more sequence divergence is expected. Therefore, structure alignment was performed on each pair of representative PDB structures from the hit and the domain model using the jCE algorithm [52] (see Figure 8, HMMER2/3 alignments are available at the associated WWW site [37]).

The model AAA (PF00004.24, row 6) is a family of ATPases associated with various cellular activities. The ATP-dependent metal binding core of the domain’s representative PDB structure (1LV7|A) consists of the characteristic Walker A or P-loop motif, Walker B motif and sensor motif, each extending beyond a ß-strand [53]. The hit CHLI_PORPU (representative structure PDB:1GP8|A) from plant is a magnesium chelatase that is involved in chlorophyll biosynthesis. Its ATP core also consists of the three hallmark motifs (Walker A/B and sensor motifs) [54]. Although the total HMM’s E-values between the hit and model were insignificant at 1.8 (HMMER2) and 1.1e-1, 5.9 (HMMER3), the fold-relevant E-values were nevertheless significant at 2.2e-6 (HMMER2) and 3.1e-3, 1.9e-4 (HMMER3). In contrast, the remnant segments’ E-values were large at 1.4e + 2 (HMMER2) and 3.1e + 2, 1.4e + 5 (HMMER3). Independently, a structural alignment revealed that, despite vast differences between the loop lengths of the two structures, a reasonable RMSD score of 3.91 over an alignment length of 255 positions was achievable over the structural elements (See Figure 5A). The ATP binding domains of both hit CHLI_PORPU and model AAA are indeed homologous.

Next, the model adh_short (PF00106.20, row 7) is a family of NADP-dependent oxidoreductases. Its representative PDB structure (3MJC|A) is an A-type ketoreductases consisting of two subdomains, a N-terminal sub-structural domain and a C-terminal catalytic subdomain that binds NADP^+^ and its ß-ketoacyl substrates [55]. On the other hand, the hit HEM1_METKA (pdb: 1GPJ|A) is a glutamyl-tRNA reductase which essential for initiating tetrapyrrole biosynthesis in plants and prokaryotes. Structurally, it consists of 3 domains : a N-terminal RNA-binding domain, a NADPH-binding domain and dimerization domain [56]. The standard E-values of hit to model were insignificant at 0.17 for HMMER2 but significant at 7.9e-6 for HMMER3 over a small fragmented piece. However, both fold-related E-values were significant (HMMER2: 1.1e-5, HMMER3: 1.5e-9) while both remnant segments’ E-values were insignificant (HMMER2: 9.0e-1, HMMER3: 5.3e + 5). Separately, a structural alignment between the two PDB structures gave a good RMSD score of 3.52 over 188 alignment positions between the 3MJC structure and the NADPH-binding domain of 1GPJ (See Figure 5B). Again, the structural alignment revealed the major differences in the loop lengths. Nevertheless, both hit and domain share a homologous NADP^+^/NADPH binding structure.

Finally, the model Form_Nir_trans (PF01226.12, row 8) describes the multi-membrane formate/nitrite transporter (PDB: 3KCU|E) of bacteria that facilitates the formate/nitrite transport essential for anaerobic respiration [57]. On the other hand, the hit TIP12_MAIZE is a plant aquaporin (representative structure PDB: 1YMG|A) that transport water and small neutral solute across the membrane [58]. Interestingly, it has been previously reported that the fold of the formate transporter is uncannily similar to the family of aquaporins despite a low sequence identity of 9-12% [59], thus raising the question if this transporter is indeed a channel. Consistent with previous findings, the structural alignment between the two representative structures produced a good RMSD of 3.2 over 273 alignment positions (See Figure 5C). Meanwhile, the hit TIP12_MAIZE was only detectable by the HMMER2 domain model at an insignificant standard E-value of 0.13, but its fold-relevant segments’ E-value was nevertheless significant at 1.2e-4. Interestingly, its remnant segments’ E-value also showed high significance at 9.4e-5. The latter suggests that, like the diverse family of GPCRs where the loop regions confers the sub-family functions [60,61], a similar role might also be expected with the non-fold-related segments in the formate/nitrite/aquaporin family.

Taken together, we have illustrated that the dissection framework provides the segment-based scores (e.g. the fold-related and other segments’ scores) for a more concise assessment of sequence similarity as evidence for homology. To emphasize, filtering of compositionally-bias sequence segment might be unnecessary since false hits will be occluded under this framework when their non-fold-related segments appeared significant statistically. Most importantly, the framework provides an opportunity to elucidate the obscured true hits hidden among the false ones in the twilight E-value range of 0.1 to 10.

### Quality score as a proxy to identify the structural segments of domain models for score dissection

In an ideal situation, the combined PDB/DSSP data provides the best information for dissecting a domain model into its fold-related and remaining segments for score reconstruction. But currently, only a small portion of domain models have an associated PDB structure. As such, one needs a surrogate for estimating the potentially more conserved elements and remaining segments for the dissection framework to be applicable on a larger set of domain models.

For this purpose, the alignment quality measure (called quality score further in the text) that assesses sequence conservation in CLUSTALX [62] was investigated; yet, the exact form of the measure is not critical for us here. For example, one could have relied on the measure used in Jalview [63,64] or others [65,66]. As a trend, fold-critical segments will deliver dense parts in multiple alignments and, thus, generate high quality scores. In contrast, variable loops and man non-globular types of sequence will result in poor multiple alignments and, hence, produce low quality scores. As is illustrated by Figure 5, the segmentation based on DSSP annotation will, as a trend, correctly estimate fold-relevant segments (or underestimate them) whereas the score based on alignment quality tends to segment more generously including also other segments besides the most fold-relevant ones. Nevertheless, in the subsequent section, we show that the high-quality alignment segments (representative for fold-critical segments) still contain significantly higher fractions of residues engaged in secondary structural elements compared with low-quality alignment segments (representative for fold-irrelevant segments).

First, the quality score per position for each domain alignment in SMART and Pfam were computed (see equations (10, 11, 12, 13, 14 and 15) in the Methods). Alignments with less than 5 sequences were not considered for the analysis due to insufficient statistical power at a significance level of *α* = 0.05. Next, each alignment position is classified as high or low-quality based on the appropriate thresholds (see equations (16,17) in Methods). The quality score thresholds are at least 0.06 (false positive rate 5%, true positive rate 90%; see Methods section “Determination of domain-wise score cutoffs …” and the table therein) and 0.14 (false positive rate 5%, true positive rate 91%; see Methods section “Determination of domain-wise score cutoffs …” and the table therein) for SMART and Pfam domains respectively. Finally, the high-quality and low-quality segments per domain alignment were derived using equation (18).

Separately, matching structures were searched for. The global-mode HMM models were built using the HMMER2 software suite to maximize for full coverage. The HMM models were searched against the PDB FASTA sequences to obtain the most significant hit (E-value at least 0.1) with the associated secondary structure residues resolved for each alignment position using the DSSP annotations [40], and the number of structural residues (carrying “H, B, E, G, I, T, S” labels in the DSSP files) is computed.

In total, 635 (out of 808) SMART and 5876 (out of 14831) Pfam domains were able to retrieve a significant PDB hit that covers the model’s full length. Each of these domain models was then subjected to the Fisher’s exact test (see equation (9) in the Methods section “Fisher’s exact (one-tailed) test …” and also the table therein) to determine if there is an enrichment of structural residues in the high-quality segments against the low-quality segments. Interestingly, at a significance level of *α* = 0.05, 537 (out of 635) SMART domains and 4771 (out of 5876) Pfam domains were enriched with structural residues in their respective high-quality segments (find lists of domains at the associated WWW site [37]). This is more than 80% of the testable SMART/Pfam domains. For the remaining 98 SMART domains and 1105 Pfam domains, there is insufficient statistical power to reject the null hypotheses. This is supported by a control test where the same 635 SMART and 5876 Pfam domains were tested in the opposite direction to see if there is an enrichment of non-structural residues in the high-quality segments against the low-quality segments but none was significant. Thus as a trend, high-quality alignment segments and secondary structures go hand in hand. At the same time, we emphasize that secondary structural elements, as a rule, will lead to high-quality alignments, the opposite is not necessarily true; non-globular segments might also produce high-quality alignments, especially if the number of sequences is not large. For our purpose, it is enough to have the quality score as necessary condition for fold-related segments.

It is also noteworthy to mention that the dissection framework using the quality score were applied to the 13 validated examples from the preceding section. The conclusions were similar to that of Table 1 except for the cases of aquaporin TIP12_MAIZE and HEM1_METKA for HMMER2 (see Additional file 2: Table S1). In addition, the results of the SEG-derived dissection (see equations (24 and 25)) for the 13 examples were also included; a method that find low-complexity regions as surrogate for long loops and intrinsically unstructured segments [67]. Based on the SEG-derived HMMER2 results, the conclusions were generally comparable to that of the quality score. However, the SEG-derived HMMER3 results suffered from a handful of differences and inaccuracies e.g. 5 false hits of the Lipoprotein5 (PF01298) domain were concluded as true hits. From this mini study, it appears that any inaccuracy in the segmentation of domain models into its fold-critical and remnant components will be amplified in the final dissection results. This is especially true for the fragmented HMMER3 alignments. From the case study examples, the quality score is a better surrogate of PDB/DSSP information for domain model dissection when compared to the SEG-derived ones.

### The dissection framework validates the seed sequences in domain alignments and systematically identifies the potential false positive and false negative hits in HMMER searches

In this section, the behavior of the score dissection framework when applied to the hits’ alignments returned by domain models from HMMER searches was examined. For this purpose, 285 (out of 537) SMART and 2381 (out of 4771) Pfam domain models were taken from the preceding section after filtering domain models with low-quality segments of less than 10 alignment positions. Furthermore, to avoid potential bias in outcome due to differences in search sensitivity by either HMMER2 or HMMER3, both were used for the generation of the initial result sets where only common hits by both HMMER2 and HMMER3 were dissected.

First, each selected SMART/Pfam domain was searched (via HMMER2/3 hmmsearch) against (i) the seed database consisting of seed sequences and (ii) UniProt/SwissProt database to generate altogether 4 sets of scores: HMMER2/seed, HMMER3/seed, HMMER2/SwissProt and HMMER3/SwissProt score sets. Next, for each score set, the hit alignments were dissected into high-quality (enriched with structural residues) and low-quality segments. The corresponding sub-scores as well as the total score were statistically evaluated in terms of E-values independently for both high- and low-quality parts (see Table 2 and the Methods section “Classification of hits in the comparative HMMER2 and HMMER3 analysis”).

**Table 2:** Label of individual HMMER hits (TP, FN, FP, TN) based on E-values of total score, high-quality score and low-quality score

**Type**	**E-value**		
	**Total score**	**High-quality score**	**Low-quality score**
TN	> 0.1	> 0.1	> 0.1
	> 0.1	> 0.1	≤ 0.1
TP	≤ 0.1	≤ 0.1	> 0.1
	≤ 0.1	≤ 0.1	≤ 0.1
FP	≤ 0.1	> 0.1	≤ 0.1
FN	> 0.1	≤ 0.1	> 0.1
?	> 0.1	≤ 0.1	≤ 0.1
	≤ 0.1	> 0.1	> 0.1

Type (?) occurs when the score threshold causes the total score to become insignificant (despite significant high and low-quality score) or vice versa.

In the statistical evaluation, the E-values were calculated using a standard database size of 540261 (UniProt as of April 2013; using equation (2) in the Methods section). This implies that the HMMER3 E-values were adjusted since their original E-values were computed based on the size of the returned set. On the other hand, the peculiarity in HMMER2 E-value calculation previously reported in [20] (jumping between two statistics) was suppressed and the usage of the extreme value distribution (EVD) was enforced in all computations. Finally, the significance call for E-value is set at 0.1 as recommended by the HMMER authors [38]. Subsequently, all hits were tagged as true-positive (TP), false-negative (FN), true-negative (TN) and false-positive (FP) (see Table 2).

**Figure 9:**
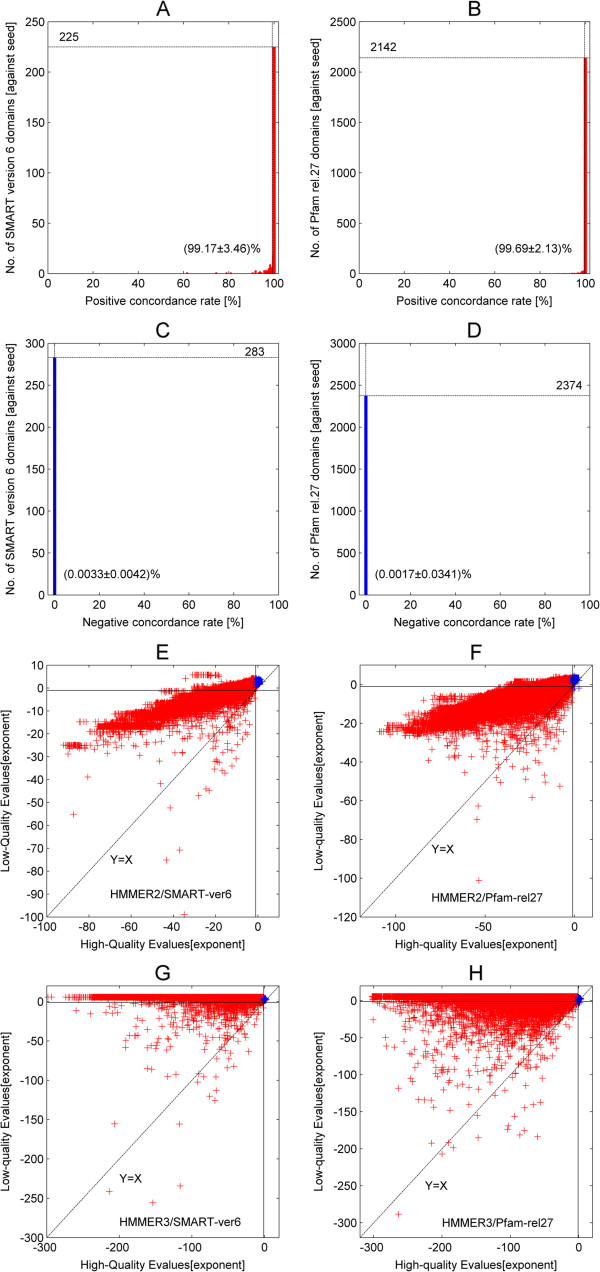
**Histograms of the positive and negative concordance rates when applied to seed sequences of 285 SMART and 2381 Pfam domain models. High-quality E-values versus low-quality E-values plots for concordance hits from HMMER2 and HMMER3-dissected results.** Figure **A** and **B** depict the histograms of the positive concordance rates for the 285 SMART and 2381 Pfam domain models respectively. On average, the positive concordance rates are (99.17 ± 3.46)% for SMART and (99.69 ± 2.13)% for Pfam, suggesting that almost all the seed sequences were correctly labeled as true hits (see vertical dotted lines). 225 (out of 285) SMART and 2142 (out of 2381) Pfam domains have a 100% positive concordance rate as depicted by the horizontal dotted lines. Likewise, Figure **C** and **D** show the histograms of the negative concordance rates for the same sets of domains. On average, the SMART and Pfam domains have a negative concordance rate of (0.0033 ± 0.0042)% and (0.0017 ± 0.0341)% respectively (see vertical dotted lines), implying that almost none of the seed sequences are mistaken as false hits. 283 (out of 285) SMART and 2374 (out of 2381) Pfam domains have a zero negative concordance rate as marked by the horizontal dotted lines. Figure **E** and **F** plot the high-quality E-values versus the low-quality E-values of the positive (in red) and negative (in blue) concordance hits of the HMMER2/SMART and HMMER2/Pfam dissected results respectively. Similarly, Figure **G** and **H** show similar plots for HMMER3/SMART and HMMER3/Pfam dissected results respectively.

Finally, the hits from HMMER2/seed and HMMER3/seed score sets were paired as long as the hits shared a common sequence segment to create a unified set between HMMER2 and HMMER3 results. The same was repeated for HMMER2/SwissProt and HMMER3/SwissProt score sets. Among the paired hits, they can be sub-classified into the concordance and discordance class. Accordingly, the concordance class contains hit results agreed upon by both HMMER2 and HMMER3 where the positive concordance class suggests that the hits are true while the negative concordance class suggests that the hits are false. On the other hand, the discordance class contains the results where HMMER2 and HMMER3 disagreed upon. Fundamentally, this class arises due to the differences in model parameterization and search/alignment algorithm attributed by the two flavors of HMMER. It is beyond the scope of this work to resolve which version of HMMER is better suited for the purpose. In addition, unmatched or orphaned hits are also excluded since this touches on the issue of search sensitivity and it is again not the focus of this work on score dissection (see Table 3 and the Methods section “Classification of hits in the comparative HMMER2 and HMMER3 analysis”).Figure 9 shows the base performance of the dissection framework when applied on the seed score set. Basically, one would expect a high positive concordance rate (an ideal value of 100%) and a low negative concordance rate (an ideal value of 0%) per domain model given that all its seed sequences are considered to be true hits. This also necessarily follows that the high-quality scores/E-values are more dominant than the low-quality counterparts for these seed sequences.Figure 9A and B depict the histograms of the positive concordance rates (see equation (19) in Methods) for the 285 SMART and 2381 Pfam domain models respectively. Note that the total paired hits included the discordance hits. Generally speaking, 225 (out of 285) SMART and 2142 (out of 2381) Pfam domains under investigation exhibit a perfect positive concordance rate as depicted by the horizontal dotted lines. On average, the positive concordance rate was (99.17 ± 3.46)% for SMART and (99.69 ± 2.13)% for Pfam as depicted by the vertical dotted lines. This suggests that almost all the seed sequences were correctly labeled as true hits.

**Table 3:** Classification of paired/orphaned hits for comparative HMMER2 and HMMER3 analysis

**Group**	**Classification**	**Type**
Paired hits	*HMMER*2^*TRUE*^*HMMER*3^*TRUE*^ (Positive concordance)	TPTP
		TPFN
		FNTP
		FNFN
	*HMMER*2^*FALSE*^*HMMER*3^*FALSE*^ (Negative concordance)	TNTN
		FPTN
		TNFP
		FPFP
	*HMMER*2^*TRUE*^*HMMER*3^*FALSE*^ (Discordance type 1)	TPFP
		FNFP
		TPTN
		FNTN
	*HMMER*2^*FALSE*^*HMMER*3^*TRUE*^ (Discordance type 2)	FPTP
		FPFN
		TNTP
		TNFN
Orphaned hits	*HMMER*2^*ONLY*^	TP
		FN
		FP
		TN
	*HMMER*3^*ONLY*^	TP
		FN
		FP
		TN

However, there were about a dozen of domains that have deviated from the ideal rate of 100% quite significantly. At below 90% positive concordance rate, there were altogether 9 Pfam and 4 SMART domains. A detailed breakdown of the seed sequence classification of these 13 domains was given in Table 4. Among these domains, the discordance rates of several domains like SM00185 (ARM), PF10590.4 (PNPOx_C_seed), SM00733 (Mterf), SM00304 (HAMP), PF00433.19 (Pkinase_C) and PF13894.1 (zf-C2H2_4) stood out at more than 20% (20.99%, 21.41%, 25.16%, 38.76%, 45.18% and 71.43% respectively). Incidentally, their domain lengths range between 49 and 159 alignment positions (on average about 100 alignment positions). This implies that for these short domains, an E-value threshold of 0.1 is not optimal.

**Table 4:** Detail breakdown of the seed sequence classification of 9 Pfam and 4 SMART domains with positive concordance rate of < 90%

**Pfam/SMART domains**	**Domain length**	**Positive concordance/Total discordance**	**Total common hits**	**Orphaned hits HMMER2/3**	**Positive concordance (%)**	**Total discordance (%)**
PF00433.19 Pkinase_C	159	108/89	197	55/0	54.82	45.18
PF01426.13 BAH	349	53/10	63	4/0	84.13	15.87
PF02098.11 His_binding	296	19/4	23	0/0	82.61	17.39
PF02965.12 Met_synt_B12	309	14/2	16	0/0	87.50	12.50
PF05594.9 Fil_haemagg	160	122/16	138	17/0	88.41	11.59
PF10590.4 PNPOx_C_seed	112	268/73	341	0/0	78.59	21.41
PF11736.3 DUF3299	235	79/13	92	0/0	85.87	14.13
PF13894.1 zf-C2H2_4	105	2/5	7	577/0	28.57	71.43
PF15612.1 WHIM1	66	29/4	33	3/0	87.88	12.12
SM00185 ARM	66	128/34	162	7/0	79.01	20.99
SM00304 HAMP	122	79/50	129	91/0	61.24	38.76
SM00320 WD40	119	580/137	717	1055/0	80.89	19.11
SM00733 Mterf	49	115/39	155	90/0	74.19	25.16

There was also another interesting observation with regard to the differences in search sensitivity between the HMMER variants. For the cases of SM00320 (WD40) and PF13894.1 (zf-C2H2_4), it was found that the number of orphaned hits found by HMMER2 only (see column 5 in Table 4) was more than the number of common hits that can be paired between HMMER2 and HMMER3 (see column 4; Table 4). As a side effect, they suffered a low positive-concordance rate. An investigation on their domain model revealed that more than half the alignment positions are made up by gaps rather than sequences (see supplementary website [37] for alignments). Thus, the list of domain models that dramatically differ from the optimal recovery rate of sequences in this test can also be seen as a suggestion for domains that might benefit from seed alignment re-valuation and polishing. This might include either alignment re-arrangement and/or exclusion of some of the seed sequences.Meanwhile, Figure 9C and D show the histograms for the negative concordance rates (see equation (20) in Methods) of the same sets of domains. In this case, 283 (out of 285) SMART and 2374 (out of 2381) Pfam domains have a zero negative concordance rate (see horizontal dotted lines). On average, the SMART and Pfam domains have a negative concordance rate of (0.0033 ± 0.0042)% and (0.0017 ± 0.0341)% respectively (see vertical dotted lines), implying that almost none of the seed sequences are mistaken as false hits. Taken together, the dissection framework has asserted the validity of the seed sequences as true hits of their respective domains.The concordance hits were also plotted in terms of their high-quality (fold-critical surrogate) E-values and low-quality (remnant surrogate) E-values in Figure 9E to H. The positive concordance hits are in red while the negative ones are in blue. Figure 9E and F shows the concordance hits generated by HMMER2 for SMART and Pfam domains. From both plots, the trend where the high-quality E-values are more dominant than the low-quality E-values is apparent (in red). This implies that these positive concordance seed sequences are indeed true hits of the respective SMART and Pfam domains. Meanwhile, a small number of negative concordance hits reside in the insignificance quadrant defined by high-quality E-value > 0.1 and low-quality E-value > 0.1. These are the hits that had contributed to the non-zero discordance rates. Meanwhile, Figure 9G and H depict the SMART/Pfam results for HMMER3. Essentially, the same conclusion can be made.

**Figure 10:**
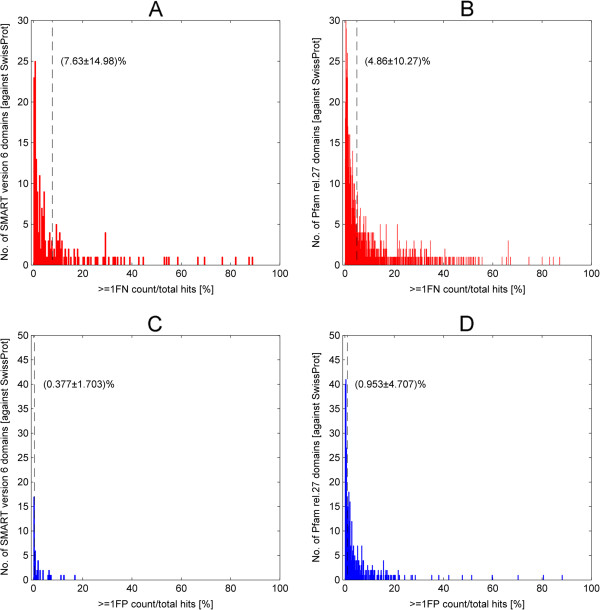
**Histograms of the false-negative and false-positive rates of 197 (out of 285) SMART and 1195 (out of 2381) Pfam domain models when applied to SwissProt/UniProt database.** Figure **A** and **B** show the histograms of 197 (out of 285) SMART and 1195 (out of 2381) Pfam domain models with non-zero FN rates respectively. The remaining 88 SMART and 1186 Pfam domains with zero FN rate were excluded from the plots. In particular, the non-zero FN rate domains potentially generated FN hits in the HMM searches. On average, the FN rates were (7.63 ± 14.98)% and (4.86 ± 10.27)% for SMART and Pfam as marked by the vertical dashed lines. Similarly, Figure **C** and **D** depict the histograms of the non-zero FP rates for 42 (out of 285) SMART and 370 (out of 2381) Pfam domains. The remaining 243 SMART and 2011 Pfam domains with zero FP rates were excluded from the plots. In contrast to the FN rates, the FP rates were relatively lower. The average FP rate for SMART is (0.377 ± 1.703)% and (0.953 ± 4.707)% for Pfam, as depicted by the vertical dashed lines. Note that all the averages were taken over 285 SMART and 2381 Pfam domains respectively.

Having established the baseline performance of the dissection framework, we then attempt to quantify the level of false-negative (FN) and false-positive (FP) hits from the results of the unified SwissProt score set generated earlier (see Figure 7). To emphasize, a FN hit is a positive hit that has been mistaken as a negative hit due to its inability to score well against the low-quality segments while a FP hit is a negative hit that is thought to be a true hit due to a significant score on the low-quality segments. The low-quality segment score is especially redundant for the current domain models under investigation since these segments harbored mostly residues which contribute lesser to the overall fold of a protein than the structural residues. As a measure of FN and FP rates, the sum of TPFN, FNTP and FNFN hits and the sum of FPTN, TNFP and FPFP over the total paired hits was taken respectively (see equations (22 and 23) in Methods and Table 3).Figure 10A and B show the histograms of the non-zero FN rates for 197 (out of 285) SMART and 1195 (out of 2381) Pfam domain models respectively. The remaining 88 SMART and 1186 Pfam domains with zero FN rates were excluded from the plots. In particular, these 197 SMART and 1195 Pfam domains potentially generated FN hits in the HMM searches. In fact, some of the FN hits from these domain models were validated as true hits like the magnesium chelatase (CHLI_PORPU) and the glutamyl-tRNA reductase (HEM1_METKA) from our earlier illustration. Henceforth, it is suggestive that there are many yet to be validated homologous relationship, albeit distant, between these FN hits and their associated domain model that requires case-to-case clarification. On average, the FN rates were (7.63 ± 14.98)% and (4.86 ± 10.27)% for SMART and Pfam respectively (see vertical dashed lines).Meanwhile, Figure 10C and D depict the histograms of the non-zero FP rates for 42 (out of 285) SMART and 370 (out of 2381) Pfam domains. The remaining 243 SMART and 2011 Pfam domains with zero FP rates were excluded from the plots. In contrast to the FN rates, the FP rates were relatively lower where the average FP rate for SMART is (0.377 ± 1.703)% and (0.953 ± 4.707)% for Pfam (see vertical dashed lines). Unsurprisingly, since most domain models were constructed from the well-curated SwissProt sequences, this resulted in only 42 SMART and 370 Pfam domains with non-zero FP rates. Indeed, the current domain models have generally very low false hits inclusion as expected. Note that all the averages above were taken over 285 SMART and 2381 Pfam domains respectively.

In hindsight, SMART and Pfam domain models have never been constructed to find all true hits (to ensure low FN rates) and this is not a matter to worry. It is more important in this context that the FP rate is extreme low (<1%) for most domain models. The few exceptional models with high FP rates deserve re-visiting and some modifications in their seed alignment. However, it is important to bear in mind that the error rates estimated here are suggestive of baseline rates since the searches have been performed over UniProt/SwissProt, which is a relatively small database. The expected error rates might be higher when a larger database such as NCBI’s non-redundant protein database is considered.

## Discussion

### Sequence homology concept in its current implementation and the necessity of dissecting sequence alignments

The sequence homology concept is backed by an inductive proof. It originates from the observation that homologous proteins share a high degree of sequence similarity, protein fold and biological function. The key to sharing a similar fold, implying a similar function, between the homologs is dependent on the similarity between the more conserved parts, most importantly the structural elements. As such, the evidence for homology should stem from the similarity between the aligned structural elements and key functional motifs with less emphasis from the other sequence segments. As we delve deeper into the search space, higher sequence divergence is to be expected and it will dilute overall sequence similarity and consequently, the homology signal. Therefore, the emphasis on similarity between the structural elements in alignments is the key to finding the homologs (both the close and the distant ones) while keeping the false ones at bay.

Despite its simplicity and elegance, the sequence homology concept is not readily computable since homology has no direct measure. It can at best be formulated into a hypothesis to be tested from the sequence similarity which is a necessary but insufficient condition for concluding homology. Although similarity by chance can be removed by some statistical criterion like E-value, often, the main issue is dealing with the statistically significant similarities of any aligned pieces (as the program outputs) that are concluded as homologous instead of convergence as alternative. Since current sequence search packages can only operate strictly in similarity space, this has a tendency to promote, to some extent, the fallacy that ‘high sequence similarity implies homology’.

Even in current times, this fallacy is still being extensively discussed by several authors, e.g. by Varshavsky and coworkers who coined the term “sequelog” in an attempt to differentiate homology from high sequence similarity [68] and by Theobald who highlighted the sins of sequence similarity derived p-values in concluding common ancestry [69]. However, there was no proposed quantitative solution on the fallacy issue. In mitigation, certain convergence cases in the form of compositional bias segments can be suppressed by pre-filtering with SEG prior to BLAST searches or by turning on ‘null2’ and ‘nobias’ options in HMMER searches, but this also comes with the price of sacrificing some sensitivity (i.e., the ability to detect true hits) [10]. On top of that, not all loop segments are compositionally-biased per se. For example, the extracellular loops of GPCR are important in functionally distinguishing the diverse GPCR families [60].

Thus, the sequence homology concept has yet to be fully implemented in current sequence homology search packages because mindful distinction between contributions from evolutionary important pieces versus spurious similarity pieces was never explicitly dealt with; hence, this necessitates for the dissection of an alignment for explicit segments to be reevaluated. As we emphasized in the Introduction, a (globular) domain is a special protein sequence unit with structural (autonomous hydrophobic core), thermodynamic (independent folding and melting) and evolutionary (domain shuffling) implications [30]. Protein domain libraries widely used for homology-based annotation contain a sizeable number of entries that do not represent domains in this sense. Thus, score dissection becomes an option to deal with this problem. As a necessary condition to be considered as a true hit, the fold-relevant segments should either be more statistically significant than the other segments or minimally be statistically significant on its own.

### The dissection framework and its implications in evaluating and detecting homology in annotation pipelines

In our proposed dissection framework, an alignment is dissected into its high-quality segments (representing fold-relevant residues) and low-quality segments (representing other residues) with the subsequent purpose of statistically evaluating the two segment-based score sums. Together with the original scores/E-values, these segment-based sums provide a new level of granularity to the dissection framework for determining if a hit is true (true-positive and false-negative) or false (true-negative and false-positive). In a nutshell, the dissection framework has created a new paradigm in which homology can be evaluated more concisely and, at the same time, more faithful to the sequence homology concept. And for the purist of the homology concept, sequence searches now have a better chance to escape the fallacy of ‘high sequence similarity implies homology’.

For the true-positives of the domain model, the dissection framework can reassert their validity as legit hits with respect to the domain. Indeed, when the framework was applied to the seed sequences of 285 SMART and 2381 Pfam domain models (with PDB/DSSP information; selected based on enriched structural residues in their high-quality segments), they exhibited the average positive and negative concordance rates of 99% and almost 0% respectively. These results imply that the seed sequences were recognized correctly by the framework as true hits of the domains.

On the other hand, cases of false hits (false-positives and true-negatives) will be occluded by the framework due to their significant low-quality scores/E-values. This scenario was played out by the case study of the 10 false hits (IF2P_HUMAN, IF2P_MOUSE, IF2P_PONAB, NUCL1_ORYSJ, MLL2_MOUSE, CORTO_DROME, DHKL_DICDI, AMOT_MOUSE, NUCL_HUMAN, PK4_DICDI) where their original HMMER2 E-values were insignificant yet significant for HMMER3. Despite a contradictory conclusion from the HMMER variants, their remnant segment-based E-values were indisputably significant for both HMMER versions. Thus, HMMER3 hits were tagged as false-positives while the same hits by HMMER2 were labeled as true-negatives. In both cases, they were considered as false hits by the framework. Interestingly, pre-filtering of compositionally-bias sequence segment may become less critical under the dissection framework since these hits will anyhow exit as false hits due to their significant remnant segments’ E-values. This also meant that the ‘null2 model correction’ and the ‘nobias’ option in HMMER2/3 can be turned off to maximize for search sensitivity to allow more hits.

Given the results in this work, a quantitative criterion for assessing segmented HMM scores in annotation pipelines might include the expectation (i) for the fold-relevant contribution resulting in a low E-value (e.g., <0.01 or <0.001) independently of the E-value for the total alignment and/or (ii) for the ratio between the E-value of the fold-critical part versus that of the remnant contribution clearly below 1.

To emphasize, score dissection with regard to fold-critical and other segments is a generic concept that can be applied to any sequence or multiple aligment comparison technique. This idea can be easily extended, for example, to the BLAST-based approach with minor adaptations: first, the extraction of the EVD parameters from the blast statistics and second, the parameters used for score reconstruction need to be extracted from BLOSUM/PAM for BlastP algorithm and PSSM for PSI-Blast algorithm.

Most importantly, the dissection itself should aim squarely at approximating the location of globular domains by applying either tertiary structure finding algorithms or any tools for detecting non-globular segments. We can only warn against applying non-physical, non-evolutionary dissection principles such as cutting sequences arithmetically first in two parts, then in four and then, maybe, in eight as many might be tempted to. This approach is likely to distribute fold-critical residues to many of the segments, hence diluting evolutionary information instead of enriching it in one class.

### The dissection approach helps finding yet unexplored homology relationships

Perhaps, the most interesting additional capability of the dissection framework, aside from being able to isolate false hits, is its proposal of unexplored homologous relationships between the hits and domain models. This means the recovery of hits presently being falsely labeled as negatives. When the dissection framework was applied to the search results against UniProt/SwissProt for these 285 SMART and 2381 Pfam domains, it revealed an overall average false-positive rate of less than 1% but the average false-negative rates of 7.63% for SMART and 4.86% for Pfam. Although the low false-positive rate implies that the current domain models have generally very low false hits inclusion, the moderate false-negative rates suggest that there are many potential true hits that are obscured by bad E-values. This situation was exemplified by our case study where the previously insignificant true hits (CHLI_PORPU, HEM1_METKA, TIP12_MAIZE) were obscured as a result of heavy score penalties on the low-quality alignment segments. However, they were subsequently rescued by their significant fold related segments’ E-values.

In particular, the discovery of the homologous relationship between the plant aquaporin (TIP12_MAIZE) and formate/nitrate transporter (PF01226.12), which indicates that the latter is actually a channel, was essentially exclusive to the structure-alignment based approaches. Even though certain sequence search methods might detect some level of sequence similarity between aquaporin and formate transporter but their E-values remain statistically insignificant (e.g. the HHPred server [36] returns E-value of 20 between aquaporin and formate transporter). However, with the proposed dissection framework, this evolutionary relationship can be rediscovered in sequence similarity space through the justification of a statistically significant fold-critical E-value. Taken together, we have shown that it is possible to explore deeper into sequence space to recover novel true hits without admitting the false ones. Surprisingly, this is achievable without tweaking or modifying the existing search algorithms but by simply performing postmortem dissection of alignments and re-evaluation of the segment-based scores.

### Estimation of evolutionary segments in domain models

It is neither practical nor reasonable to create domain models without their non-fold-related segments so identifying these pieces is a matter of necessity. A critical component in the proposed dissection framework is the pre-definition of the evolutionary-related pieces in the domain models. The PDB/DSSP data gave the best delineation of fold-critical segments from the remaining ones. However, it suffices only as a proof of concept for the dissection framework and is not readily applicable to domain models that do not have a significant PDB structure representation. Hence, a more generalized measure is required as a reasonable surrogate for estimating structural segments of domain models. As such, the quality score from CLUSTALX [62] as representative of similar alignment quality scales, which measures sequence conservation for each alignment column, was investigated.

As it turns out, the Fishers’ exact test showed that 537 SMART and 4771 Pfam domains were enriched with structural residues in their respective high-quality segments. This was out of 635 SMART and 5876 Pfam domains with a representative PDB structure. Correspondingly, the high-quality and low-quality segments were able to reasonably estimate the fold-critical and remaining segments respectively. This was further reinforced when the examples from the case study were reexamined by the dissection framework using the quality score instead of PDB/DSSP. Overall, the conclusions were similar with the exception of 2 hits (TIP12_MAIZE, HEM1_METKA for HMMER2 results). For the cases of these 2 hits, this signifies that quality score is an overestimate of fold-critical segments and as a result, it tends to underestimate the false-negative hits by adding part of the negative remnant sum to the fold-critical sum. Indeed, a scrutiny on the high-quality segments of the associated domain models for these 2 hits revealed that some of these segments were covered by loop residues when compared against the PDB/DSSP annotations.

In hindsight though, one should err on the side of conservativeness; i,e., one needs to be more stringent with claiming a true hit. Therefore, the quality-score is still a reasonable estimate for partitioning the fold-relevant and remnant segments. Nevertheless, one can easily add more estimates like low-complexity/disorder predictors (SEG [67], IUPred [70], GlobPlot [71], tools for predicting regions with certain posttranslational modifications and translocation signals [72,73], etc.) on top of the existing quality score measure so that a more comprehensive definition of fold- and domain function-critical versus other segments can be derived.

However, this task of selecting/combining predictors to mimic the PDB/DSSP information to perform domain segmentation is not straightforward. When compared to the quality-score results, the application of SEG-based dissection to the 13 case study examples worked equally well for the HMMER2 hits but less so for many of the fragmented HMMER3 hits. This revealed the sub-optimality of SEG in elucidating the fold-critical domain segments when compared to the quality-score. Consequently, the effect is more pronounced in the short fragmented HMMER3 hits than the longer HMMER2 hits. Despite so, the SEG-derived segments can still help to identify well-conserved low-complexity segments (to be marked as remnant segments) that will otherwise be missed by the quality-score. Hence some combination of the two predictors makes sense.

In any case, the creation of a catalogue of segmentations for existing protein domain libraries such as Pfam or SMART will be necessary in the absence of complete PDB/DSSP information for a foreseeable future and it will be considered in our future work.

## Conclusions

As sequence homology can only be concluded inductively and overall sequence similarity is a measurable, necessary but insufficient criterion to justify homology, additional considerations are required to decide about homology relationships between biomolecular sequences. To distinguish the true cases from the false background might be possible in a manual study for individual cases; yet, a computerized pipeline for large-scale annotation requires quantitative conditions.

The complex hydrophobic/hydrophilic sequence pattern necessary for fold formation and conserved during evolution can be used for this purpose by dissecting the similarity score into fold-critical contributions and other parts originating from non-globular segments, long loops, etc. This work serves as a proof of concept for this idea. The dissection framework and the software tools provided with this article are useful for systematically suppressing otherwise generated false-positive hits in sequence similarity searches.

The dissection approach allows also extracting more value out of existing protein domain model databases without the need to re-edit them simply by defining segmental contribution and, thus enhancing or deemphasizing certain parts of the seed alignments.

Surprisingly, this approach was also successful in recovering hitherto hidden homology relationships by stripping away the noise created by score contributions from non-fold-critical, non-globular protein regions.

## Methods

### Reconstruction of HMMER scores and E-values

Generally speaking, the log-odd score of an alignment *v* between the HMM hidden sequence *X* and an observed hit sequence *Y* of length *L* can be re-computed by summing up a set of emission, transition and a fixed score *f*. The general equation for the total score of an alignment, where *e*_*HMM*_, *t*_*HMM*_ and *e*_*null*_, *t*_*null*_ are the emission and transition parameters of the hidden and null model respectively, is given as:

$$v=\log_{2}\frac{P\left(Y,X;e_{\mathrm{HMM}},t_{\mathrm{HMM}}\right)}{P\left(Y,X;e_{\mathrm{null}},t_{\mathrm{null}}\right)}+f$$

$$=\log_{2}\left[\frac{\prod_{i=0}^{L}P\left(Y_{i}|X_{i};e_{\mathrm{HMM}}\right)}{\prod_{i=0}^{L}P\left(Y_{i}|X_{i};e_{\mathrm{null}}\right)}\times \frac{P\left(X_{0};t_{\mathrm{HMM}}\right)\prod_{i=1}^{L}P\left(X_{i}|X_{i-1};t_{\mathrm{HMM}}\right)}{P\left(X_{0};t_{\mathrm{null}}\right)\prod_{i=1}^{L}P\left(X_{i}|X_{i-1};t_{\mathrm{null}}\right)}\right]+f$$

$$\begin{array}{l} =\sum_{i=0}^{L}\log_{2}\frac{P\left(Y_{i}|X_{i};e_{\mathrm{HMM}}\right)}{P\left(Y_{i}|X_{i};e_{\mathrm{null}}\right)}+\sum_{i=1}^{L}\log_{2}\frac{P\left(X_{i}|X_{i-1};e_{\mathrm{HMM}}\right)}{P\left(X_{i}|X_{i-1};e_{\mathrm{null}}\right)}\\ \;+\log_{2}\frac{P\left(X_{0};t_{\mathrm{HMM}}\right)}{P\left(X_{0};t_{\mathrm{null}}\right)}+f \end{array}$$

(1) $$=\sum_{i=0}^{L}\log_{2}e\left(Y_{i}|X_{i}\right)+\sum_{i=1}^{L}\log_{2}t\left(X_{i}|X_{i-1}\right)+\log_{2}t\left(X_{0}\right)+f$$

The respective transition and emission (match or insert state) score for each position can be retrieved from the respective HMM model file (created by hmmbuild). In the case of HMMER3 model files, we added an additional step to convert them to HMMER2 format (via hmmconvert −2) prior to the reconstruction step. Note that the fixed score is independent of the alignment and it is essentially constant for the same domain model. The fixed score is made up of the additional special transition scores (N- > B, N- > N, E- > C, E- > J, C- > T, C- > C, J- > B, J- > J) and annotated in ‘XT’ line of the model file.

For the computation of E-value, the maximum Gumbel extreme value distribution is used and is given as:

$$E=N\cdot P_{\mathrm{EVD}}\left(S\geq v\right)$$

(2) $$=N\cdot \left(1-{e^{-e}}^{-\lambda \left(v-\mu \right)}\right)$$

where *N* is the size of the database that was searched against, (*μ*, *λ*) are the summary statistics of the HMM domain model file (‘EVD’ line for HMMER2, ‘STATS LOCAL FORWARD’ line for HMMER3).

For the creation of the domain models, the following command and options were used:

(HMMER2) hmmbuild -F --amino --fast --gapmax 1

hmmcalibrate --seed 0 --num 5000

(HMMER3) hmmbuild --amino --fast --symfrac 0.0

hmmconvert −2

For searching domain models against sequence databases, the following command and options were used:

(HMMER2) hmmsearch --null2 -E 10

(HMMER3) hmmsearch --nonull2 --nobias -E 10

As an initial consideration, the ‘null2 correction model’ and the ‘nobias’ options were turned off since (i) it was unclear how these penalties were calculated and on which part of the alignment, particularly for HMMER3, and (ii) it improves search sensitivity according to the manuals [38,39].

### Regression and fit

Here, the linear relationship *W* = *v* is tested to affirm the reproducibility of the HMMER scores. For each domain, a linear regression (without intercept) is performed between a set of original scores *v* and reconstructed scores *W* for each domain (with *P* hits) and the associated slope $\hat{\beta }$ and the coefficient of determination *r*^2^ is computed.

It is important to note that the regression will be performed on a set of seed sequences’ scores per domain. Therefore, it is inevitable that these scores would cluster closely. As such, an extra point at the origin (i.e. 0,0) is added to each set of scores to alleviate the bias towards the high scores. For a set of scores that is well spread, the additional point has little impact.

The slope $\hat{\beta }$ is given as:

(3) $$\hat{\beta }=\frac{\sum_{i=1}^{P}w_{i}v_{i}}{\sum_{i=1}^{P}v_{i}^{2}}$$

The coefficient of determination *r*^2^ is given as:

(4) $$r^{2}=\frac{{\left(\sum_{i=1}^{P}v_{i}w_{i}-\frac{\sum_{i=1}^{P}v_{i}\sum_{i=1}^{P}w_{i}}{P}\right)}^{2}/\sum_{i=1}^{P}v_{i}^{2}-\frac{{\left(\sum_{i=1}^{P}v_{i}\right)}^{2}}{P}}{\sum_{i=1}^{P}w_{i}^{2}-{\left(\sum_{i=1}^{P}w_{i}\right)}^{2}/P}$$

### Derivation of error estimates model

With respect to a given domain model, an alignment between the HMM emitted sequence and the hit sequence can be recomputed by summing the appropriate emission, transition and fixed scores taken from the HMMER2/3 model parameters. This reconstructed score *W* can be subjected to (i) rounding errors, (ii) parameter conversion estimation and (iii) unavailability of local model parameters ((ii) and (iii) applies to HMMER3 hmmconvert, see also Figure 1). Here, an error model *ϵ* can be derive to quantify the approximation error where $\epsilon \sim N\left(\mu_{\epsilon },\sigma_{\epsilon }^{2}\right)$ for each given domain model. Collectively, the reconstructed score *W* is related to the original score *v* by:

(5) W=v+ϵ

It follows that the mean and variance of the component-wise error model *ϵ* are given as:

(6) $$\mu_{\epsilon }=\frac{1}{P}\sum_{i=1}^{P}\left(w_{i}-v_{i}\right)$$

(7) $$\sigma_{\epsilon }^{2}=\frac{1}{P}\sum_{i=1}^{P}{\left[\left(w_{i}-v_{i}\right)-\frac{1}{P}\sum_{i=1}^{P}\left(w_{i}-v_{i}\right)\right]}^{2}$$

for *P* pairs of original and reconstructed scores.

As a measure against the representative domain score, the error estimate can be written as a relative measure given as:

(8) $$\epsilon_{r}=\frac{\mu_{\epsilon }}{\mu_{v}}$$

where the representative domain score is estimated by $\mu_{v}=\frac{1}{P}\sum_{i=1}^{P}v_{i}$.

### Fisher’s exact (one-tailed) test for structural/loop residues in high-quality versus low-quality segments in domain alignment

First consider an alignment between a HMMER sequence and a hit sequence with its associated DSSP annotations. Then, let the DSSP structure residue be denoted by a set *R*_*S*_ = {*H*, *B*, *E*, *G*, *I*, *T*, *S*} where H = alpha helix, B = residue in isolated beta-bridge, E = extended strand that participates in beta ladder, G = 3-helix (3/10 helix), I = 5 helix (pi helix), T = hydrogen bonded turn and S = bend. On the other hand, let the unstructured set be denoted by *R*_*U*_ = {'',−} where ‘’ and – represent loop residue and alignment gap respectively. Furthermore, let the total high-quality and low-quality residue counts be *R*_1_ and *R*_2_ respectively while the total structure and non-structural residue counts be *C*_1_ and *C*_2_ respectively (See Table 5). The total count of all residues is *N*. As such, the null hypothesis is stated as:

**Table 5:** 2-by-2 contingency table setup for Fishers’ exact test

	**Outcome**		
	**#{H,B,E,G,I,T,S}**	**#{",−}**	
High-quality residues	*f*_11_	*f*_12_	*R*_1_
Low-quality residues	*f*_21_	*f*_22_	*R*_2_
	*C*_1_	*C*_2_	N

*H*_0_: The proportion of high-quality residues containing structure residues *R*_*s*_ is no greater than the low-quality residues containing structure residues *R*_*s*_.

Consequently, the p-value to be tested at a significance level of *α* = 0.05 is evaluated via the hypergeometric cumulative density function in the following form:

(9) $$P\left(X>f_{11}\right)=1-P\left(X\;\leq \;f_{11}\right)$$

where $P\left(X=f_{11}\right)=\left(\begin{array}{c} R_{1}\\ f_{11} \end{array}\right)\left(\begin{array}{c} N-R_{1}\\ C_{1}-f_{11} \end{array}\right)/\left(\begin{array}{c} N\\ C_{1} \end{array}\right)$

### Domain quality score

We use the alignment quality measure as adapted from CLUSTALX [62]. The domain quality score can be calculated for each column in the sequence alignment to measure the consensus level of amino acid per alignment position. Suppose we have an alignment of amino acid residues *a* of *M* sequences with *N* positions. This can be expressed as:

$$\begin{array}{ccccc} a_{11} & a_{12} & a_{13} & .......... & a_{1N}\\ a_{21} & a_{22} & a_{23} & .......... & a_{2N}\\ . & & & & \\ . & & & & \\ a_{M1} & a_{M2} & a_{M3} & .......... & a_{\mathrm{MN}} \end{array}$$

The consensus vector for column *j* over *R* amino acid residues *a* = {1, 2, 3, …, *R*} is written as:

(10) $$\begin{array}{l} X_{j}=\frac{1}{M}{\left[\begin{array}{c} F_{1j}\\ F_{2j}\\ \ddots \\ F_{\mathrm{Rj}} \end{array}\right]}^{T}\left[\begin{array}{cccc} c_{11} & c_{12} & \cdots & c_{1R}\\ c_{21} & c_{22} & & c_{2R}\\ \ddots & \ddots & \ddots & \ddots \\ c_{R1} & c_{R2} & \cdots & c_{\mathrm{RR}} \end{array}\right]\\ \;=\left[\begin{array}{cccc} X_{1j} & X_{2j} & \cdots & X_{\mathrm{Rj}} \end{array}\right] \end{array}$$

where *F*_*rj*_ is the count of residue *r* in column *j*, *c*_*rt*_ is the score (taken from BLOSUM62 matrices) of between residue *r* and residue *t*. At the same time, the score vector of residue *a*_*ij*_ for sequence *i* at position *j* over *R* residues is given as:

$$S_{\mathrm{ij}}=\left[\begin{array}{cccc} c_{1a_{\mathrm{ij}}} & c_{2a_{\mathrm{ij}}} & \cdots & c_{Ra_{\mathrm{ij}}} \end{array}\right]$$

For each sequence *i* and position *j*, the distance measure between the consensus column *j* and the residue *a*_*ij*_ over *R* residues is then given as:

(11) $$D_{\mathrm{ij}}=\sqrt{\sum_{r=1}^{R}\left(X_{\mathrm{rj}}-c_{ra_{\mathrm{ij}}}\right)}$$

Finally, the quality score, *Q* for column *j* over *M* sequences is given as:

(12) $$Q_{j}=\frac{\sum_{i=1}^{M}D_{\mathrm{ij}}}{M}$$

Since quality score *Q* as a distance measure is expected to be near zero for high consensus while large for low consensus, it would be more intuitive to invert and limit the range of *Q* as follows:

(13) $${\hat{Q}}_{j}=1-\frac{Q_{j}-\min\left\{Q_{1},Q_{2},\cdots ,Q_{N}\right\}}{\max\left\{Q_{1},Q_{2},\cdots ,Q_{N}\right\}-\min\left\{Q_{1},Q_{2},\cdots ,Q_{N}\right\}},\;0\leq \hat{Q}\leq 1$$

Finally, inverted quality score ${\hat{Q}}_{j}$ for column *j* is normalized by multiplying the ratio of amino acids (less gaps) over the total sequences given as:

(14) $${\tilde{Q}}_{j}=\frac{k}{M}\times {\hat{Q}}_{j}$$

where *k* is the count of valid amino acid residues.

### Minimum number of sequences in an alignment

Given an alignment, for each position, let *M* be number of sequences (excluding gaps in the particular column) and let *k* be the sum of Bernoulli random variable I (an indicator variable). The indicator variable emits either a value of one for a positive prediction or zero for a negative prediction. Collectively, this can be written as the Binomial random variable.

(15) $$P\left(X\;\geq \;k\right)=\sum_{x\geq k}^{M}\left(\begin{array}{c} M\\ x \end{array}\right)p^{x}{\left(1-p\right)}^{M-x}$$

Under equal chance condition, the null and alternate hypotheses are stated as *H*_*o*_ : *p* ≤ 0.5, *H*_*A*_ : *p* > 0.5 to be tested at a significance level of *α* = 0.05. Under this setup, the minimum number of sequences per alignment position is determined to be at least 5 since there is insufficient power to reject the null hypothesis for sequences below 4. This is because the smallest p-values for *M* = 4 is *P*(*X* ≥ 4) = 0.0625, *M* = 3 is *P*(*X* ≥ 3) = 0.125, *M* = 2 is *P*(*X* ≥ 2) = 0.25 and *M* = 1 is *P*(*X* ≥ 1) = 0.5. All these p-values are larger than the significance level of *α* = 0.05.

### Determination of domain-wise quality score cutoff for low and high-quality segment

Here, the appropriate cutoff to declare if a quality score is high or low is determined. With respect to a domain alignment, (i) the quality score per position and (ii) the number of valid amino acids per position ignoring gaps are first determined. Then, each quality score per position is classified into the following two classes: (i) if the alignment column has less than 5 valid amino acids and (ii) if alignment column has at least 5 or more amino acids.

**Figure 11:**
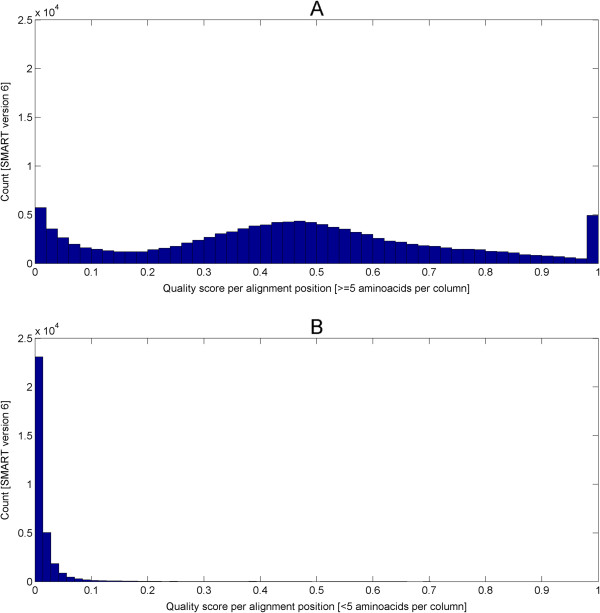
**The distributions of the two classes of quality score for SMART version 6.** Figure **A** depicts the quality scores alignment positions of 5 or more amino acids. It is a trimodal distribution, most likely, arising from low-quality scores from weak alignments (left peak), average-quality scores from the typical alignments (center peak) and high-quality scores from homogenous alignments (right peak). In contrast, Figure **B** shows mostly the low quality scores from weaker alignment positions of less than 5 valid amino acids.

The distributions of the two classes of quality score for SMART (version 6) is shown in Figure 11. Figure 11A (quality scores for 5 or more amino acids) depicts an interesting trimodal distribution, most likely, arising from 3 unique distributions of low-quality scores from weak alignments (left peak), average-quality scores from the typical alignments (center peak) and high-quality scores from homogenous alignments (right peak). In contrast to Figure 11B, it is apparent that the lower quality scores mainly originate from alignment positions with less than 5 valid amino acids which are indicative of weak alignment segments. Conservatively speaking, the latter distribution forms the minimal negative set or the null hypothesis. To select the desired false-positive rate (FPR) and true-positive rates (TPR) for subsequent application, the quality score cutoff is permuted from 0 to 1 and tabulated in Table 6. Based on the table, the FPR of 5% corresponds to a quality score of at least 0.06 and renders a TPR of 90%. Note that FPR and TPR are given as:

**Table 6:** Error rates (false-positive and true-positive rates) of quality scores at various quality score cutoffs for SMART (version 6)

**Cutoff**	**TP**	**FN**	**FP**	**TN**	**FPR**	**TPR**
0.01	113960	3217	12650	19966	0.38785	0.973
0.02	111450	5727	6610	26006	0.20266	0.951
0.03	109530	7653	4157	28459	0.12745	0.935
0.04	107900	9277	2813	29803	0.08625	0.921
0.05	106480	10702	2070	30546	0.06347	0.909
0.06	105260	11919	1608	31008	0.04930	0.898
0.10	101690	15491	789	31827	0.02419	0.868
0.20	95355	21823	294	32322	0.00901	0.814
0.30	86126	31052	169	32447	0.00518	0.735
0.40	69734	47444	72	32544	0.00221	0.595
0.50	48713	68465	47	32569	0.00144	0.416
0.60	31278	85900	15	32601	0.00046	0.267
0.70	20413	96765	1	32615	0.00003	0.174
0.80	12727	104450	0	32616	0.00000	0.109
0.90	7473	109710	0	32616	0.00000	0.064

(16) TPR=TPTP+FN

(17) FPR=FPFP+TN

**Figure 12:**
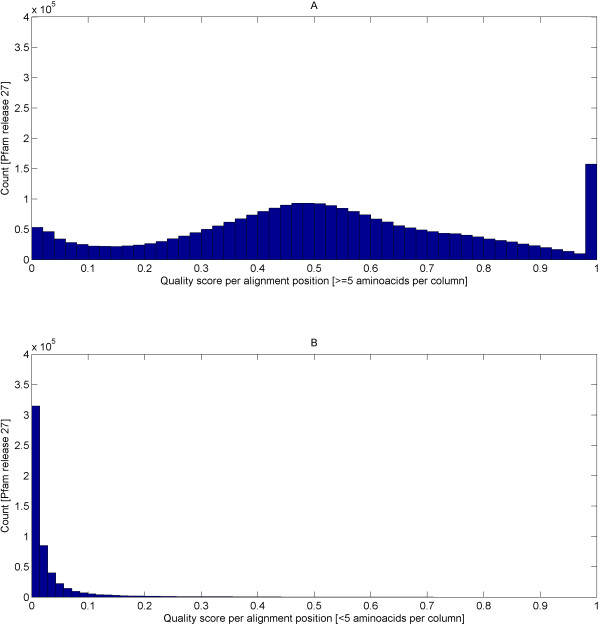
**The distributions of the two classes of quality score for Pfam release 27.** Compared to the distributions from SMART (version 6), Figure **A** exhibits the same trimodal distribution while Figure **B** also depicts mainly the lower quality scores from weaker alignment positions with less than 5 amino acids.

Similarly, the same procedure was performed on Pfam (release 27). In a similar fashion, Figure 12A exhibits the same trimodal distribution while Figure 12B once again depicts that the low-quality scores originates from alignment positions with less than 5 amino acids or sparsely aligned segments. Table 7 gives the respective error rates (FPR, TPR) for various quality score cutoff. Based on the table, the FPR of 5% corresponds to a quality score of at least 0.14 and renders a TPR of 91%.

**Table 7:** Error rates (false-positive and true-positive rates) of quality scores at various quality score cutoffs for Pfam (release 27)

**Cutoff**	**TP**	**FN**	**FP**	**TN**	**FPR**	**TPR**
0.01	2479900	21831	265240	267000	0.49835	0.991
0.05	2384300	117450	79402	452830	0.14919	0.953
0.10	2314800	186960	38684	493550	0.07268	0.925
0.12	2292300	209440	31629	500610	0.05943	0.916
0.13	2281400	220350	28938	503300	0.05437	0.912
0.14	2270400	231360	26412	505820	0.04963	0.908
0.15	2259500	242240	24371	507860	0.04579	0.903
0.20	2201800	299960	16844	515390	0.03165	0.880
0.30	2027300	474450	8670	523570	0.01629	0.810
0.40	1718400	783320	4060	528180	0.00763	0.687
0.50	1277700	1224000	1990	530250	0.00374	0.511
0.60	857990	1643800	978	531260	0.00184	0.343
0.70	571700	1930100	21	532210	0.00004	0.229
0.80	361280	2140500	0	532240	0.00000	0.144
0.90	217480	2284300	0	532240	0.00000	0.087

Consequently, we are interested to find segments in a domain alignment of length *N*. Hence each segment can be written in set notation such that:

(18) $$A=\left\{a_{k},a_{k+1},a_{k+2},\ldots ,a_{N}\right\},\;a_{k}\in A,\;a_{k+1}-a_{k}=1$$

where ${\tilde{Q}}_{a_{k}}<\mathrm{cutoff}$ (for low-quality segment) or ${\tilde{Q}}_{a_{k}}\geq \mathrm{cutoff}$ (high-quality segment)

### Classification of hits in the comparative HMMER2 and HMMER3 analysis

In the proposed comparative analysis, the hits are first generated from both HMMER2 and HMMER3 using the same domain alignment and searched against a common database (e.g. UniProt). In addition, only hits with E-value of 0.1 and below (as suggested by Sean Eddy in his original HMMER2 manual) are considered.

Using this E-value criterion, one can then define each hit (whether HMMER2 or 3) as true positive (TP), false negative (FN), true negative (TN) and false positive (FP) based on the E-values of its total score, high-quality segment score and low-quality segment score. Essentially, the TP and FN hits belong to a positive set while the FP and TN hits belongs to a negative set.

The type of hits and associated conditions are listed in Table 2. For completeness sake, undefined type (?) has been included. The latter can occur when the fixed score causes the total score to become insignificant (despite significant high and low-quality score) or vice versa. In practice, these cases are almost non-existing.

Consequently, the intersection of HMMER2 and HMMER3 hits will result in mainly two large groups: a paired group and an orphaned group. To elaborate, a paired hit is a hit covering the same sequence segment by both HMMER2 and HMMER3. An orphaned hit is (i) a hit scored on the same sequence but non-overlapping segments by HMMER2 and HMMER3; or (ii) a hit covered by either HMMER2 or HMMER3 only.

In the paired group, one can further sub-divide the HMMER2/3 hits into four classes of (i) positive concordance hits where both HMMER2/3 mark the hits as positive, (ii) negative concordance hits where both HMMEr2/3 mark the hits as negative (iii) discordance type 1 where HMMER2 marks the hits as positive but HMMER3 marks them as negative and (iv) discordance type 2 hits where HMMER2 marks the hits as negative but HMMER3 marks them as positive. The orphaned groups contain mutually exclusive hits that are found by either HMMER2 or HMMER3. See Table 3 for details. As such, the positive and negative concordance rates are given as:

(19) $$\mathrm{PositiveConcordance}=\frac{\mathrm{TPTP}+\mathrm{TPFN}+\mathrm{FNTP}+\mathrm{FNFN}}{\mathrm{coun}t_{\mathrm{Pairedhits}}}$$

(20) $$\mathrm{NegativeConcordance}=\frac{\mathrm{TNTN}+\mathrm{FPTN}+\mathrm{TNFP}+\mathrm{FPFP}}{\mathrm{coun}t_{\mathrm{Pairedhits}}}$$

(21) $$\mathrm{TotalDiscordance}=\frac{\mathrm{discordanceType}1+\mathrm{discordanceType}2}{\mathrm{coun}t_{\mathrm{Pairedhits}}}$$

Meanwhile, classes that contain the FN and FP hits are of high interest in this work. A FN hit is a positive hit that has been obscured due to a need to score an alignment for the low-quality segment while a FP hit is a negative hit that has been carried over to significance due to the high-scoring low-quality segments. To quantify the false-negative and false-positive rates in a given domain model, the formulas are given as:

(22) $$\mathrm{FNrate}=\frac{\geq 1\mathrm{FN}}{\mathrm{coun}t_{\mathrm{Pairedhits}}}=\frac{\mathrm{TPFN}+\mathrm{FNTP}+\mathrm{FNFN}}{\mathrm{coun}t_{\mathrm{Pairedhits}}}$$

(23) $$\mathrm{FPrate}=\frac{\geq 1\mathrm{FP}}{\mathrm{coun}t_{\mathrm{Pairedhits}}}=\frac{\mathrm{TPFN}+\mathrm{FNTP}+\mathrm{FNFNF}=}{\mathrm{coun}t_{\mathrm{Pairedhits}}}$$

### SEG-derived domain model probabilities and high/low-complexity segments

For each seed sequence in a domain alignment, the gaps were first removed and then predicted using the SEG low-complexity sequence predictor [67] with the following parameters: windows size = 25, lower cutoff = 2.9 and upper cutoff = 3.2.

If a residue is flagged as low-complexity by SEG, then its corresponding position in the domain alignment is marked as 0 to indicate a negative prediction, otherwise, it takes a value of 1 to indicate a positive prediction. Essentially, each column in the alignment will be marked by 1’s or 0’s and can be viewed as a sum of Bernoulli random variables. Then to test for the significance of positive predictions in each alignment column, a p-value (see equation (15)) is calculated and tested at a significance level of 0.05. If the null hypothesis is rejected, the expected positive prediction count *k*_exp_ is calculated as:

(24) $$k_{\text{exp}}=P\left(X\geq k\right)\times k$$

Otherwise, *k*_exp_ is set to zero. Finally, the per-column probability indicating that the consensus column (with *M* sequences) is representative of a high-complexity residue (or fold-critical surrogate) is given as:

(25) pexp=0.01ifkexp=0kexpMifotherwise

Consequently, the SEG-derived segments of the domain alignment can be obtained via equation (18) at a cutoff of 0.8 (i.e. *p*_exp_ ≥ *cutoff* implies high-complexity while *p*_exp_ < *cutoff* implies low-complexity).

## Competing interests

The authors declare that they have no competing interests.

## Authors’ contributions

WCW and FE conceived and designed the experiments: WCW implemented the software and performed the experiments. WCW, BE and FE analyzed the data. WCW, SMS, BE and FE wrote, read and approved the final manuscript.

## Supplementary Material

Additional file 1 **Zip-archive of the software DissectHMMER.** This archive contains all files to create a program executable for dissecting the score for a given HMMER2/3 protein domain model – query sequence alignment. Click here for file

Additional file 2: Table S1 This table contains the examples of validated false hits from 5 Pfam domains (*PF01298.13 Lipoprotein5, PF04814.8 HNF-1 N, PF05134.8 T2SL, PF09110.6 HAND, PF10390.4 ELL*) and validated true hits from 3 Pfam domains (*PF00004.24 AAA, PF00106.20 adh_short, PF01226.12 Form_Nir_trans*). The segmentation of domain models is based on the alignment quality score. The data presented is complementary to Table 1 in the main text. Click here for file

## References

[B1] Bork P Dandekar T Diaz-Lazcoz Y Eisenhaber F Huynen M Yuan Y Predicting function: from genes to genomes and back J Mol Biol 1998 283 707 725 10.1006/jmbi.1998.2144 9790834

[B2] Bork P Gibson TJ Applying motif and profile searches Methods Enzymol 1996 266 162 184 8743684 10.1016/s0076-6879(96)66013-3

[B3] Doolittle RF Blombach E Amino-acid sequence investigations of fibrinopeptides from various mammals: evolutionary implications Nature 1964 202 147 152 10.1038/202147a0 14156289

[B4] Fitch WM Margoliash E Construction of phylogenetic trees: a method based on mutational distances as estimated from cytochrome c sequences is of general applicability Science 1967 155 279 284 10.1126/science.155.3760.279 5334057

[B5] Dayhoff MO Computer analysis of protein evolution Sci Am 1969 221 86 95 5789703 10.1038/scientificamerican0769-86

[B6] Jardine N Van Rijsbergen CJ Jardine CJ Evolutionary rates and the inference of evolutionary tree forms Nature 1969 224 185 10.1038/224185a0

[B7] Brew K Vanaman TC Hill RL Comparison of the amino acid sequence of bovine alpha-lactalbumin and hens egg white lysozyme J Biol Chem 1967 242 3747 3749 6038502

[B8] Allen SC Acharya KR Palmer KA Shapiro R Vallee BL Scheraga HA A comparison of the predicted and X-ray structures of angiogenin. Implications for further studies of model building of homologous proteins J Protein Chem 1994 13 649 658 10.1007/BF01890464 7702747

[B9] Palmer KA Scheraga HA Riordan JF Vallee BL A preliminary three-dimensional structure of angiogenin Proc Natl Acad Sci U S A 1986 83 1965 1969 10.1073/pnas.83.7.1965 3457369 PMC323210

[B10] Wong WC Maurer-Stroh S Eisenhaber F More than 1,001 problems with protein domain databases: transmembrane regions, signal peptides and the issue of sequence homology PLoS Comput Biol 2010 6 e1000867 10.1371/journal.pcbi.1000867 20686689 PMC2912341

[B11] Doolittle RF Similar amino acid sequences: chance or common ancestry? Science 1981 214 149 159 10.1126/science.7280687 7280687

[B12] Doolittle RF Similar amino acid sequences revisited Trends Biochem Sci 1989 14 244 245 10.1016/0968-0004(89)90055-8 2773041

[B13] Reeck GR De Haën C Teller DC Doolittle RF Fitch WM Dickerson RE Chambon P McLachlan AD Margoliash E Jukes TH “Homology” in proteins and nucleic acids: a terminology muddle and a way out of it Cell 1987 50 667 10.1016/0092-8674(87)90322-9 3621342

[B14] Altschul SF Madden TL Schaffer AA Zhang J Zhang Z Miller W Lipman DJ Gapped BLAST and PSI-BLAST: a new generation of protein database search programs Nucleic Acids Res 1997 25 3389 3402 10.1093/nar/25.17.3389 9254694 PMC146917

[B15] Eddy SR What is a hidden Markov model? Nat Biotechnol 2004 22 1315 1316 10.1038/nbt1004-1315 15470472

[B16] Eddy SR A probabilistic model of local sequence alignment that simplifies statistical significance estimation PLoS Comput Biol 2008 4 e1000069 10.1371/journal.pcbi.1000069 18516236 PMC2396288

[B17] Eisenhaber B Eisenhaber F Subramaniam S Sequence complexity of proteins and its significance in annotation “Bioinformatics” in the Encyclopedia of Genetics, Genomics, Proteomics and Bioinformatics. Volume 4 2005 1 New York: Wiley Interscience doi:10.1002/047001153X.g403313

[B18] Eisenhaber B Eisenhaber F Posttranslational modifications and subcellular localization signals: indicators of sequence regions without inherent 3D structure? Curr Protein Pept Sci 2007 8 197 203 10.2174/138920307780363424 17430201

[B19] Wong WC Maurer-Stroh S Eisenhaber F Not all transmembrane helices are born equal: towards the extension of the sequence homology concept to membrane proteins Biol Direct 2011 6 57 10.1186/1745-6150-6-57 22024092 PMC3217874

[B20] Wong WC Maurer-Stroh S Eisenhaber F The Janus-faced E-values of HMMER2: extreme value distribution or logistic function? J Bioinform Comput Biol 2011 9 179 206 10.1142/S0219720011005264 21328712

[B21] Wong WC Maurer-Stroh S Schneider G Eisenhaber F Transmembrane helix: simple or complex Nucleic Acids Res 2012 40 W370 W375 10.1093/nar/gks379 22564899 PMC3394259

[B22] Dickens NJ Ponting CP THoR: a tool for domain discovery and curation of multiple alignments Genome Biol 2003 4 R52 10.1186/gb-2003-4-8-r52 12914660 PMC193644

[B23] Letunic I Doerks T Bork P SMART 6: recent updates and new developments Nucleic Acids Res 2009 37 D229 D232 10.1093/nar/gkn808 18978020 PMC2686533

[B24] Schultz J Milpetz F Bork P Ponting CP SMART, a simple modular architecture research tool: identification of signaling domains Proc Natl Acad Sci U S A 1998 95 5857 5864 10.1073/pnas.95.11.5857 9600884 PMC34487

[B25] Finn RD Mistry J Tate J Coggill P Heger A Pollington JE Gavin OL Gunasekaran P Ceric G Forslund K The Pfam protein families database Nucleic Acids Res 2010 38 D211 D222 10.1093/nar/gkp985 19920124 PMC2808889

[B26] Sammut SJ Finn RD Bateman A Pfam 10 years on: 10,000 families and still growing Brief Bioinform 2008 9 210 219 10.1093/bib/bbn010 18344544

[B27] Gilks WR Audit B De Angelis D Tsoka S Ouzounis CA Modeling the percolation of annotation errors in a database of protein sequences Bioinformatics 2002 18 1641 1649 10.1093/bioinformatics/18.12.1641 12490449

[B28] Gilks WR Audit B De Angelis D Tsoka S Ouzounis CA Percolation of annotation errors through hierarchically structured protein sequence databases Math Biosci 2005 193 223 234 10.1016/j.mbs.2004.08.001 15748731

[B29] Ouzounis CA Karp PD The past, present and future of genome-wide re-annotation Genome Biol 2002 3 COMMENT2001 11864365 10.1186/gb-2002-3-2-comment2001 PMC139008

[B30] Eisenhaber F Bork P Schomburg D Sequence and Structure of Proteins Recombinant Proteins, Monoclonal Antibodies and Theraeutic Genes 1998 2 Weinheim: Wiley-VCH 43 86

[B31] Plewczynski D Rychlewski L Ye Y Jaroszewski L Godzik A Integrated web service for improving alignment quality based on segments comparison BMC Bioinforma 2004 5 98 10.1186/1471-2105-5-98 PMC497040 15271224

[B32] Ohlson T Aggarwal V Elofsson A MacCallum RM Improved alignment quality by combining evolutionary information, predicted secondary structure and self-organizing maps BMC Bioinforma 2006 7 357 10.1186/1471-2105-7-357 PMC1562450 16869963

[B33] Lin HN Notredame C Chang JM Sung TY Hsu WL Improving the alignment quality of consistency based aligners with an evaluation function using synonymous protein words PLoS One 2011 6 e27872 10.1371/journal.pone.0027872 22163274 PMC3229492

[B34] Henikoff JG Greene EA Taylor N Henikoff S Pietrokovski S Using the blocks database to recognize functional domains Curr Protoc Bioinformatics 2002 Chapter 2 Unit 18792933 10.1002/0471250953.bi0202s00

[B35] Jaroszewski L Li Z Cai XH Weber C Godzik A FFAS server: novel features and applications Nucleic Acids Res 2011 39 W38 W44 10.1093/nar/gkr441 21715387 PMC3125803

[B36] Soding J Biegert A Lupas AN The HHpred interactive server for protein homology detection and structure prediction Nucleic Acids Res 2005 33 W244 W248 10.1093/nar/gki408 15980461 PMC1160169

[B37] Wong WC Maurer-Stroh S Eisenhaber B Eisenhaber F HMM score dissection website [http://mendel.bii.a-star.edu.sg/SEQUENCES/ProblemDomains-HMMscore-dissection/]

[B38] Eddy S HMMER User’s Guide Version 2.3.2 2003

[B39] Eddy S HMMER User’s Guide Version 3.0rc1 2010

[B40] Secondary structure files - RCSB Protein Data Bank - RCSB PDB [http://www.rcsb.org/pdb/files/ss.txt]

[B41] Ooi HS Kwo CY Wildpaner M Sirota FL Eisenhaber B Maurer-Stroh S Wong WC Schleiffer A Eisenhaber F Schneider G ANNIE: integrated de novo protein sequence annotation Nucleic Acids Res 2009 37 W435 W440 10.1093/nar/gkp254 19389726 PMC2703921

[B42] Eisenhaber F Eisenhaber F Prediction of Protein Function: Two Basic Concepts and One Practical Recipe Discovering Biomolecular Mechanisms with Computational Biology 2006 1 Georgetown and New York: Landes Biosciences and Springer 39 54

[B43] Schneider G Sherman W Kuchibhatla D Ooi HS Sirota FL Maurer-Stroh S Eisenhaber B Eisenhaber F Trajanoski Z Protein sequence-structure-function-network links discovered with the ANNOTATOR software suite: application to Elys/Mel-28 Computational Medicine 2012 1 Vienna: Springer 111 143

[B44] Noinaj N Easley NC Oke M Mizuno N Gumbart J Boura E Steere AN Zak O Aisen P Tajkhorshid E Structural basis for iron piracy by pathogenic Neisseria Nature 2012 483 53 58 10.1038/nature10823 22327295 PMC3292680

[B45] Dephoure N Zhou C Villen J Beausoleil SA Bakalarski CE Elledge SJ Gygi SP A quantitative atlas of mitotic phosphorylation Proc Natl Acad Sci U S A 2008 105 10762 10767 10.1073/pnas.0805139105 18669648 PMC2504835

[B46] Grune T Brzeski J Eberharter A Clapier CR Corona DF Becker PB Muller CW Crystal structure and functional analysis of a nucleosome recognition module of the remodeling factor ISWI Mol Cell 2003 12 449 460 10.1016/S1097-2765(03)00273-9 14536084

[B47] Chi YI Frantz JD Oh BC Hansen L Dhe-Paganon S Shoelson SE Diabetes mutations delineate an atypical POU domain in HNF-1alpha Mol Cell 2002 10 1129 1137 10.1016/S1097-2765(02)00704-9 12453420

[B48] Abendroth J Bagdasarian M Sandkvist M Hol WG The structure of the cytoplasmic domain of EpsL, an inner membrane component of the type II secretion system of Vibrio cholerae: an unusual member of the actin-like ATPase superfamily J Mol Biol 2004 344 619 633 10.1016/j.jmb.2004.09.062 15533433

[B49] Levchenko T Aase K Troyanovsky B Bratt A Holmgren L Loss of responsiveness to chemotactic factors by deletion of the C-terminal protein interaction site of angiomotin J Cell Sci 2003 116 3803 3810 10.1242/jcs.00694 12902404

[B50] Troyanovsky B Levchenko T Mansson G Matvijenko O Holmgren L Angiomotin: an angiostatin binding protein that regulates endothelial cell migration and tube formation J Cell Biol 2001 152 1247 1254 10.1083/jcb.152.6.1247 11257124 PMC2199208

[B51] Banks CA Kong SE Spahr H Florens L Martin-Brown S Washburn MP Conaway JW Mushegian A Conaway RC Identification and Characterization of a Schizosaccharomyces pombe RNA Polymerase II Elongation Factor with Similarity to the Metazoan Transcription Factor ELL J Biol Chem 2007 282 5761 5769 10.1074/jbc.M610393200 17150956

[B52] Shindyalov IN Bourne PE Protein structure alignment by incremental combinatorial extension (CE) of the optimal path Protein Eng 1998 11 739 747 10.1093/protein/11.9.739 9796821

[B53] Krzywda S Brzozowski AM Verma C Karata K Ogura T Wilkinson AJ The crystal structure of the AAA domain of the ATP-dependent protease FtsH of Escherichia coli at 1.5 A resolution Structure 2002 10 1073 1083 10.1016/S0969-2126(02)00806-7 12176385

[B54] Fodje MN Hansson A Hansson M Olsen JG Gough S Willows RD Al-Karadaghi S Interplay between an AAA module and an integrin I domain may regulate the function of magnesium chelatase J Mol Biol 2001 311 111 122 10.1006/jmbi.2001.4834 11469861

[B55] Zheng J Taylor CA Piasecki SK Keatinge-Clay AT Structural and functional analysis of A-type ketoreductases from the amphotericin modular polyketide synthase Structure 2010 18 913 922 10.1016/j.str.2010.04.015 20696392

[B56] Moser J Schubert WD Beier V Bringemeier I Jahn D Heinz DW V-shaped structure of glutamyl-tRNA reductase, the first enzyme of tRNA-dependent tetrapyrrole biosynthesis EMBO J 2001 20 6583 6590 10.1093/emboj/20.23.6583 11726494 PMC125327

[B57] Waight AB Love J Wang DN Structure and mechanism of a pentameric formate channel Nat Struct Mol Biol 2010 17 31 37 10.1038/nsmb.1740 20010838 PMC3613427

[B58] Harries WE Akhavan D Miercke LJ Khademi S Stroud RM The channel architecture of aquaporin 0 at a 2.2-A resolution Proc Natl Acad Sci U S A 2004 101 14045 14050 10.1073/pnas.0405274101 15377788 PMC521118

[B59] Theobald DL Miller C Membrane transport proteins: surprises in structural sameness Nat Struct Mol Biol 2010 17 2 3 10.1038/nsmb0110-2 20051980

[B60] Wheatley M Wootten D Conner MT Simms J Kendrick R Logan RT Poyner DR Barwell J Lifting the lid on GPCRs: the role of extracellular loops Br J Pharmacol 2012 165 1688 1703 10.1111/j.1476-5381.2011.01629.x 21864311 PMC3372823

[B61] Barwell J Woolley MJ Wheatley M Conner AC Poyner DR The role of the extracellular loops of the CGRP receptor, a family B GPCR Biochem Soc Trans 2012 40 433 437 10.1042/BST20110726 22435826

[B62] Thompson JD Gibson TJ Plewniak F Jeanmougin F Higgins DG The CLUSTAL_X windows interface: flexible strategies for multiple sequence alignment aided by quality analysis tools Nucleic Acids Res 1997 25 4876 4882 10.1093/nar/25.24.4876 9396791 PMC147148

[B63] Clamp M Cuff J Searle SM Barton GJ The Jalview Java alignment editor Bioinformatics 2004 20 426 427 10.1093/bioinformatics/btg430 14960472

[B64] Waterhouse AM Procter JB Martin DM Clamp M Barton GJ Jalview Version 2–a multiple sequence alignment editor and analysis workbench Bioinformatics 2009 25 1189 1191 10.1093/bioinformatics/btp033 19151095 PMC2672624

[B65] Yao H Mihalek I Lichtarge O Rank information: a structure-independent measure of evolutionary trace quality that improves identification of protein functional sites Proteins 2006 65 111 123 10.1002/prot.21101 16894615

[B66] Ahola V Aittokallio T Vihinen M Uusipaikka E Model-based prediction of sequence alignment quality Bioinformatics 2008 24 2165 2171 10.1093/bioinformatics/btn414 18678587

[B67] Wootton JC Federhen S Analysis of compositionally biased regions in sequence databases Methods Enzymol 1996 266 554 571 8743706 10.1016/s0076-6879(96)66035-2

[B68] Varshavsky A ‘Spalog’ and ‘sequelog’: neutral terms for spatial and sequence similarity Curr Biol 2004 14 R181 R183 10.1016/j.cub.2004.02.014 15028230

[B69] Theobald DL On universal common ancestry, sequence similarity, and phylogenetic structure: the sins of P-values and the virtues of Bayesian evidence Biol Direct 2011 6 60 10.1186/1745-6150-6-60 22114984 PMC3314578

[B70] Tompa P Dosztanyi Z Simon I Prevalent structural disorder in E. coli and S. cerevisiae proteomes J Proteome Res 2006 5 1996 2000 10.1021/pr0600881 16889422

[B71] Linding R Russell RB Neduva V Gibson TJ GlobPlot: Exploring protein sequences for globularity and disorder Nucleic Acids Res 2003 31 3701 3708 10.1093/nar/gkg519 12824398 PMC169197

[B72] Eisenhaber B Eisenhaber F Maurer-Stroh S Neuberger G Prediction of sequence signals for lipid post-translational modifications: insights from case studies Proteomics 2004 4 1614 1625 10.1002/pmic.200300781 15174131

[B73] Eisenhaber F Eisenhaber B Kubina W Maurer-Stroh S Neuberger G Schneider G Wildpaner M Prediction of lipid posttranslational modifications and localization signals from protein sequences: big-Pi, NMT and PTS1 Nucleic Acids Res 2003 31 3631 3634 10.1093/nar/gkg537 12824382 PMC168944

