# Supplementary material for: On the necessity of dissecting sequence similarity scores into segment-specific contributions for inferring protein homology, function prediction and annotation
Source: BMC Bioinformatics. 2014 Jun 2;15:166. doi: 10.1186/1471-2105-15-166 (PMC4061105; doi:10.1186/1471-2105-15-166)
Supplement: Additional file 2: Table S1 — This table contains the examples of validated false hits from 5 Pfam domains (PF01298.13 Lipoprotein5, PF04814.8 HNF-1 N, PF05134.8 T2SL, PF09110.6 HAND, PF10390.4 ELL) and validated true hits from 3 Pfam domains (PF00004.24 AAA, PF00106.20 adh_short, PF01226.12 Form_Nir_trans). The segmentation of domain models is based on the alignment quality score. The data presented is complementary to Table 1 in the main text. [file 1471-2105-15-166-S2.pdf]

Supplementary Table 1

Examples of validated false hits from 5 Pfam domains (*PF01298.13 Lipoprotein5, PF04814.8 HNF-1 N, PF05134.8 T2SL, PF09110.6 HAND, PF10390.4 ELL*) and validated true hits from 3 Pfam domains (*PF00004.24 AAA, PF00106.20 adh_short, PF01226.12 Form_Nir_trans*). The segmentation of domain models is based on quality score (QS) and SEG25 (SG). Note that the ratio is not calculated if both fold-critical and remnant E-values are insignificant (i.e. E>0.1)

**Domain name**	**Hit name**	**HMMER version**	**Total score (E-value)**	**Fold-critical score ($E\text{-value}_1$)**	**Remnant score ($E\text{-value}_2$)**	**Ratio of $E\text{-value}_1$: $E\text{-value}_2$**
PF01298.13 Lipoprotein5	1.sp|O60841|IF2P_HUMAN (Eukaryotic translational initialization factor 5B)	HMMER2^{QS}	-183.8 (3.1)	-178.2 (2.1)	44.5 (1.4e-7)	1.5e+7
		HMMER3^{QS}	30.1 (6.7e-8)	-4.8 (1.8e+5)	32.6 (1.1e-6)	1.6e+11
Domain length: 979		HMMER2^{SG}		-151.0 (2.6e-1)	-20.9 (1.6e-5)	1.6e+4
		HMMER3^{SG}		22.3 (1.4e-3)	-2.4 (4.2e+4)	3.3e-8
PDB:3V8U|B	2.sp|Q05D44|IF2P_MOUSE (Eukaryotic translational initialization factor 5B)	HMMER2^{QS}	-184.6 (3.3)	-162.4 (6.4e-1)	29.0 (4.5e-7)	1.4e+6
		HMMER3^{QS}	26.5 (8e-7)	-2.6 (4.6e+4)	40.8 (3.7e-9)	1.2e+13
		HMMER2^{SG}		-154.0 (3.2e-1)	-21.1 (1.5e-5)	2.1e+4
		HMMER3^{SG}		47.2 (4.4e-11)	-9.0 (5.4e+5)	9.2e-17
	3.sp|Q5RDE1|IF2P_PONAB (Eukaryotic translational initialization factor 5B)	HMMER2^{QS}	-185.0 (3.4)	-158.6 (4.8e-1)	15.8 (1.2e-6)	4.0e+5
		HMMER3^{QS}	28.6 (1.8e-7)	-6.4 (3.9e+5)	33.7 (5.3e-7)	7.4e+11
		HMMER2^{SG}		-142.9 (1.4e-1)	-27.5 (2.6e-5)	5.4e+3
		HMMER3^{SG}		29.2 (1.2e-5)	-9.9 (5.4e+5)	2.2e-11
	4.sp|Q7XTT4|NUCL2_ORYSJ (Nucleolin 2)	HMMER2^{QS}	-190.8 (5.2)	-133.1 (7.3e-2)	-16.7 (1.3e-5)	5.6e+3
		HMMER3^{QS}	13.2 (8.2e-3)	3.6 (6.4e+2)	25.3 (1.9e-4)	3.4e+6
		HMMER2^{SG}		-191.1 (5.1)	10.7 (1.5e-6)	3.4e+6
		HMMER3^{SG}		15.1 (2.2e-1)	-5.6 (2.8e+5)	7.9e-7
PF04814.8 HNF-1_N (Hepatocyte nuclear factor 1)	5.sp|Q6PDK2|MLL2_MOUSE (Histone-lysine N-methyltransferase 2D)	HMMER2^{QS}	70.2 (1.5)	-69.1 (1.2)	19.1 (2.5e-8)	4.8e+7
		HMMER3^{QS}	24.5 (5.1e-6)	6.7 (2.7e+2)	25.1 (6.7e-4)	4.0e+5
		HMMER2^{SG}		-68.0 (1.1)	-14.9 (2.8e-5)	3.9e+4
		HMMER3^{SG}		3.2 (3.2e+3)	7.9 (1.2e+2)	-
Domain length: 250	6.sp|P41046|CORTO_DROME (Centrosomal/chromosomal factor)	HMMER2^{QS}	-75.5 (4.4)	-75.1 (4.0)	17.5 (3.4e-8)	1.2e+8
PDB:1IC8|B		HMMER3^{QS}	23.0 (1.6e-5)	9.2 (4.6e+1)	19.2 (4.1e-2)	1.1e+3
		HMMER2^{SG}		-71.4 (2.1)	-5.9 (4.6e-6)	4.6e+5
		HMMER3^{SG}		2.0 (7.2e+3)	18.7 (5.8e-2)	1.2e+5
	7.sp|Q54RP6|DHKL_DICDI (Hybrid signal transduction histidine kinase L)	HMMER2^{QS}	-75.6 (4.5)	-86.5 (4.0e+1)	30.6 (2.5e-9)	1.6e+10
		HMMER3^{QS}	32.6 (1.7e-8)	17.1 (1.8e-1)	29.6 (2.8e-5)	6.4e+3
		HMMER2^{SG}		-74.6 (4.0)	-18.2 (5.3e-5)	7.6e+4
		HMMER3^{SG}		5.3 (7.2e+2)	12.8 (3.7)	-
PF05134.8 T2SL (Type II secretion system protein L)	8.sp|Q8VHG2|AMOT_MOUSE (Angiomotin)	HMMER2^{QS}	-81.4 (4.5)	-66.6 (5.1e-1)	15.4 (3.0e-6)	1.7e+5
		HMMER3^{QS}	18.2 (1.8e-5)	10.7 (7.1)	26.1 (1.5e-4)	4.7e+4
		HMMER2^{SG}		-58.9 (1.6e-1)	1.7 (2.2e-5)	7.3e+3
		HMMER3^{SG}		35.3 (2.3e-7)	1.5 (4.5e+3)	5.1e-11
Domain length: 321						
PDB:1W97|L						
PF09110.6 HAND	9.sp|P19338|NUCL_HUMAN (Nucleolin)	HMMER2^{QS}	-39.7 (2.1)	-49.1 (1.3e+1)	25.5 (6.6e-6)	2.0e+6
		HMMER3^{QS}	23.3 (2.7e-5)	6.9 (2.4e+2)	18.9 (4.7e-2)	5.1e+3

(Chromatin remodeling factor ISW1a)<br><br>PDB:2Y9Z|A		HMMER2^{SG}<br>HMMER3^{SG}		-54.3 (3.6e+1)<br>-0.5 (4.4e+4)	30.2 (2.7e-6)<br>26.3 (2.5e-4)	1.3e+7<br>1.8e+8
PF10390.4<br>ELL<br>(RNA polymerase II elongation factor)<br><br>Domain length: 139<br><br>PDB:2E5N|A	10.sp|P34103|PK4_DICDI<br>(Protein kinase 4)	HMMER2^{QS}<br>HMMER3^{QS}<br><br>HMMER2^{SG}<br>HMMER3^{SG}	-70.7(3.7e-2)<br>94.5 (2.5e-27)	-130.7 (4.6e+1)<br>3.8 (6.6e+2)<br><br>-128.3 (3.5e+1)<br>0.0 (9.2e+3)	80.5 (5.8e-10)<br>100.2 (4.1e-27)<br><br>16.8 (1.2e-6)<br>47.7 (3.4e-11)	7.9e+10<br>1.6e+29<br><br>1.8e+8<br>2.7e+14
PF00004.24<br>AAA<br>(ATPase family associated with various cellular activities)<br><br>Domain Length: 252<br><br>PDB:1LV7|A	12.sp|P51394|CHLI_PORPU<br>(Magnesium-chelatase subunit ChlI)	HMMER2^{QS}<br>HMMER3^{QS}<br>HMMER3^{QS}<br><br>HMMER2^{SG}<br>HMMER3^{SG}<br>HMMER3^{SG}	-27.2 (1.8)<br>11.3 (1.1e-1)<br>5.6 (6.4)	10.3 (7.7e-4)<br>28.4 (4.5e-5)<br>23.5 (1.4e-3)<br><br>-9.7 (4.8e-2)<br>28.4 (4.5e-5)<br>23.5 (1.4e-3)	-17.1 (2.3e-1)<br>0.00 (2.1e+4)<br>0.00 (2.1e+4)<br><br>2.1 (4.1e-3)<br>1.9 (5.7e+3)<br>0.0 (2.1e+4)	3.3e-3<br>2.1e-9<br>6.7e-8<br><br>1.2e+1<br>7.9e-9<br>6.7e-8
PF00106.20<br>adh_short<br>(Short chain dehydrogenase)<br><br>Domain length: 230<br><br>PDB:3MJC|B	13.sp|Q9UXR8|HEM1_METKA<br>(Glutamyl-tRNA reductase)	HMMER2^{QS}<br>HMMER3^{QS}<br><br>HMMER2^{SG}<br>HMMER3^{SG}	-49.7 (1.7e-1)<br>23.0 (7.9e-6)	-26.4 (8.5e-3)<br>38.1 (5.0e-8)<br><br>-29.9 (1.6e-2)<br>-2.4 (1.1e+5)	-14.5 (1.2e-3)<br>-1.4 (5.7e+4)<br><br>-7.8 (4.1e-4)<br>39.0 (2.6e-8)	7.1<br>8.8e-13<br><br>3.9e+1<br>4.2e+12
PF01226.12<br>Form_Nir_trans<br>(Formate/nitrate transporter)<br><br>Domain length: 366<br><br>PDB:3KCU|E	14.sp|Q9ATM0|TIP12_MAIZE<br>(Aquaporin TIP 1-2)	HMMER2^{QS}<br><br>HMMER2^{SG}	-109.7 (1.3e-1)	-88.8 (1.2e-2)<br><br>-92.7 (1.8e-2)	-0.3 (6.0e-7)<br><br>-0.1 (5.5e-7)	2.0e+4<br><br>3.3e+4
